# Latent Class Analysis-Derived Subphenotypes are Generalizable to Observational Cohorts of Acute Respiratory Distress Syndrome: A Prospective Study

**DOI:** 10.1136/thoraxjnl-2021-217158

**Published:** 2021-07-12

**Authors:** Pratik Sinha, Kevin L Delucchi, Yue Chen, Hanjing Zhuo, Jason Abbott, Chunxue Wang, Nancy Wickersham, J. Brennan McNeil, Alejandra Jauregui, Serena Ke, Kathryn Vessel, Antonio Gomez, Carolyn M Hendrickson, Kirsten N Kangelaris, Aartik Sarma, Aleksandra Leligdowicz, Kathleen Liu, Michael A Matthay, Lorraine B Ware, Carolyn S Calfee

**Affiliations:** 1 Division of Clinical and Translational Research, Division of Critical Care, Department of Anesthesia, Washington University School of Medicine, Saint Louis, MO; 2 Department of Psychiatry; University of California, San Francisco; San Francisco, CA; 3 Department of Medicine, Division of Pulmonary, Critical Care, Allergy and Sleep Medicine; University of California, San Francisco; San Francisco, CA; 4 Cardiovascular Research Institute; University of California, San Francisco; San Francisco, CA; 5 Division of Allergy, Pulmonary, and Critical Care Medicine, Department of Medicine, Vanderbilt University Medical Center, Nashville, TN; 6 Division of Allergy, Pulmonary, and Critical Care Medicine, Department of Medicine, University of California, Zuckerberg San Francisco General Hospital and Trauma Center; 7 Division of Hospital Medicine, Department of Medicine, University of California, San Francisco; San Francisco, CA; 8 Interdepartmental Division of Critical Care Medicine, University of Toronto, Toronto, Canada; 9 Department of Medicine, Division of Nephrology, University of California, San Francisco; San Francisco, CA; 10 Department of Anesthesia; University of California, San Francisco; San Francisco, CA; 11 Department of Pathology, Microbiology and Immunology, Vanderbilt University Medical Center, Nashville, TN

## Abstract

**Rationale:**

Using latent class analysis (LCA), two subphenotypes of acute respiratory distress syndrome (ARDS) have consistently been identified in five randomized controlled trials (RCTs), with distinct biological characteristics, divergent outcomes and differential treatment responses to randomized interventions. Their existence in unselected populations of ARDS remains unknown. We sought to identify subphenotypes in observational cohorts of ARDS using LCA.

**Methods:**

LCA was independently applied to ARDS patients from two prospective observational cohorts of patients admitted to the ICU, derived from the VALID (n=624), and EARLI (n=335) studies. Clinical and biological data were used as class-defining variables. To test for concordance with prior ARDS subphenotypes, the performance metrics of parsimonious classifier models (Interleukin-8, bicarbonate, Protein C and vasopressor-use), previously-developed in RCTs, were evaluated in EARLI and VALID with LCA-derived subphenotypes as the gold-standard.

**Results:**

A two-class model best fit the population in VALID (p=0.0010) and in EARLI (p<0.0001). Class 2 comprised 26% and 37% of the populations in VALID and EARLI respectively. Consistent with the previously described “Hyperinflammatory” subphenotype, Class 2 was characterized by higher pro-inflammatory biomarkers, acidosis and increased shock and worse clinical outcomes. The similarities between these and prior RCT-derived subphenotypes were further substantiated by the performance of the parsimonious classifier models in both cohorts (AUCs 0.92–0.94). The Hyperinflammatory subphenotype was associated with increased prevalence of chronic liver disease and neutropenia and reduced incidence of chronic obstructive pulmonary disease. Measurement of novel biomarkers showed significantly higher levels of matrix metalloproteinase-8 and markers of endothelial injury in the Hyperinflammatory subphenotype, whereas, matrix metalloproteinase-9 was significantly lower.

**Conclusion:**

Previously described subphenotypes are generalizable to unselected populations of non-trauma ARDS.

## Introduction

New approaches are needed to understand the heterogeneity of ARDS, using both clinical and biologic variables.[1] Using latent class analysis (LCA), two distinct subphenotypes have consistently been identified across five randomized controlled trials (RCTs) in acute respiratory distress syndrome (ARDS).[2–5] The ARDS subphenotypes have distinct clinical and biological characteristics and divergent clinical outcomes. The Hyperinflammatory subphenotype is associated with exaggerated inflammation (increased interleukin-6 and -8), higher levels of creatinine and bilirubin, and increased mortality. Further, in three RCTs which showed no benefit from the interventions in all enrolled patients with ARDS, differential treatment responses were observed when stratified by subphenotypes.[2–4]

To date, the identification of these specific LCA-derived ARDS subphenotypes has been limited to RCTs. It is unknown whether they are generalizable to unselected populations of ARDS patients, constituting a key knowledge gap in the field. Outcomes are known to differ between observational and clinical trial populations in ARDS, with generally increased mortality in the former.[6] The lower mortality in RCTs is primarily due to the stringent inclusion criteria and exclusion of patients with high or imminent risk of mortality or non-pulmonary organ dysfunction, such as moderate to severe liver disease.[6] It is not known whether LCA in less selected ARDS populations that include patients with malignancy, chronic organ dysfunction and immunosuppression would identify the same subphenotypes, or whether new ones would emerge. Therefore, the primary objective of this study was to use LCA to identify subphenotypes in observational cohorts of ARDS patients. To evaluate whether LCA identifies subphenotypes that were similar to those seen in prior RCTs in which differential treatment responses were observed, we recapitulated our prior approaches to LCA in this study, including using similar class-defining variables.[2–5] The similarity of the subphenotypes identified in the current study to those previously described in RCTs was evaluated using parsimonious models that classify the Hyperinflammatory subphenotype and were developed in prior RCT cohorts. Finally, given the wealth of clinical data and biospecimens available in the observational cohorts, we wanted to leverage this data to evaluate and characterise the subphenotypes in greater detail based on chronic health conditions, substance-abuse history and a novel panel of protein biomarkers.

## Methods

### Patient Population

**Figure 1.**
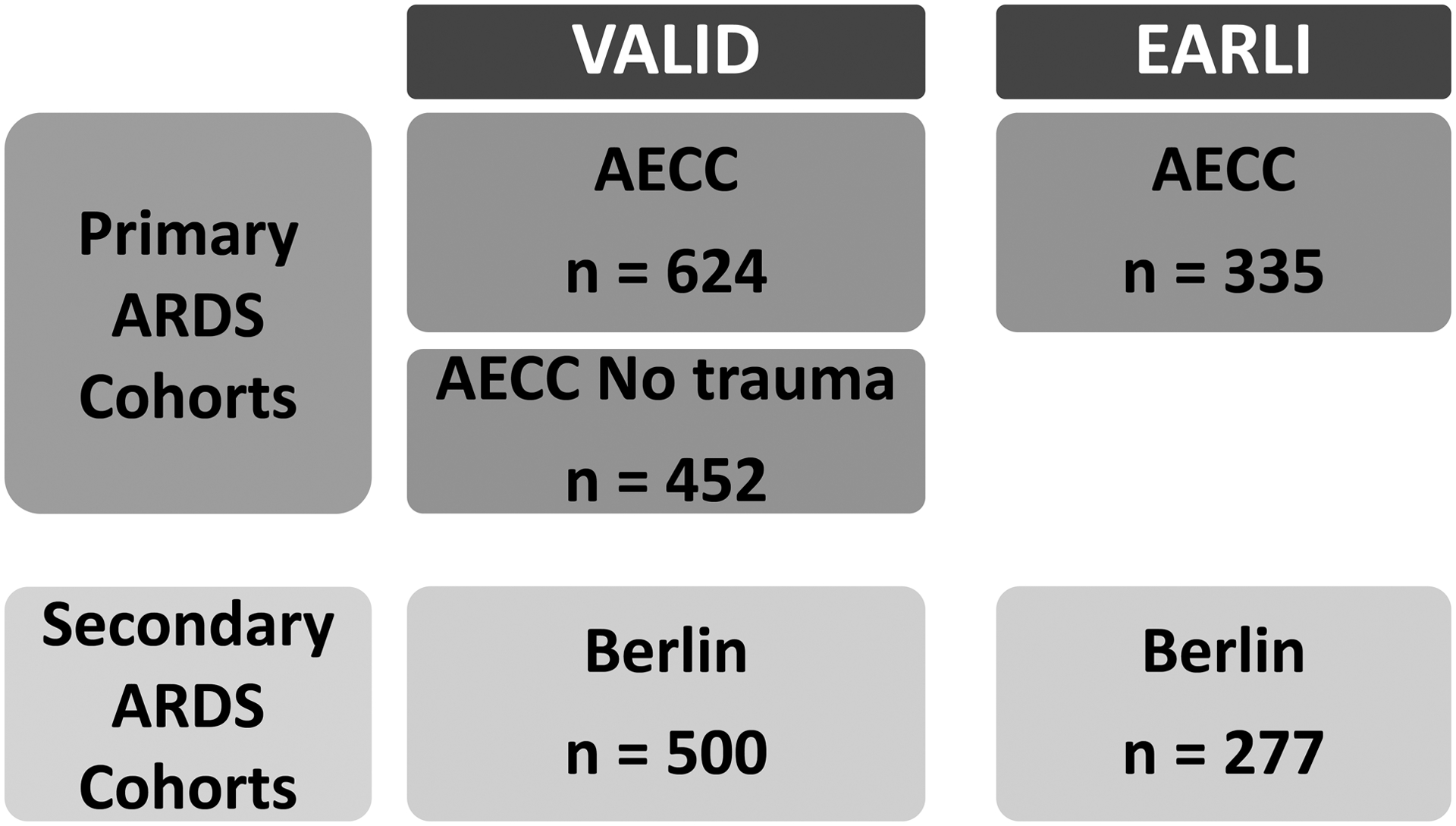
Overview of the cohorts and their subsets used for analysis in the study.

The analyses were performed on patients drawn from two distinct observational cohorts (Figure 1): Validating Acute Lung Injury markers for Diagnosis (VALID) and Early Assessment of Renal and Lung Injury (EARLI). VALID is an ongoing prospectively enrolled single-center cohort of critically-ill patients at Vanderbilt University Medical Center. Details of the study protocol have been described elsewhere.[7] Briefly, patients were enrolled into the study on the morning of day 2 of their admission in medical, surgical, trauma and cardiovascular ICUs. For the purposes of this study, patients were included for analysis if they developed ARDS on ICU day 1 or ICU day 2.

EARLI is an ongoing prospectively enrolled dual-center cohort of critically-ill patients recruited at the UCSF Medical Center and the San Francisco General Hospital. Patients are identified in the Emergency Department and are eligible for recruitment to the study if the attending physician has requested an ICU admission for the patient. The day of ICU admission constituted the first day of the study. Only patients adjudicated to have ARDS on day 1 or 2 were included for analysis. Table S1 has details of the exclusion criteria. Figure S1 depicts the screening and selection of patients in the two cohorts. These cohorts were specifically selected for analysis because they were sufficiently sized for these analyses and had the requisite biospecimens stored to analyse the biomarkers needed to recapitulate prior LCA studies.

In both studies, comprehensive demographic and clinical data including in-hospital mortality were available from the day of, or before, ARDS diagnosis. Biological samples were collected on ICU day 2 in VALID, and either in the emergency department or ICU day 1 in EARLI. Details of clinical phenotyping and ARDS adjudication can be found in the online supplement.

For the primary analysis, ARDS was defined using the American-European Consensus Conference (AECC)[8], as this allowed inclusion of patients who were not mechanically ventilated and whose enrollment pre-dated the Berlin definition. For secondary/sensitivity analyses, only patients that met the Berlin criteria for ARDS were used.[9] Finally, in the VALID cohort a post-hoc analysis was done excluding patients in whom trauma was the primary ARDS risk factor. Both studies were approved by their respective Institutional Review Boards at UCSF and Vanderbilt.

### Latent Class Analysis

Latent class analysis (LCA) was performed on each cohort using the same procedures as in prior studies.[2–5, 10] Data at, or proximal to, the time of ARDS diagnosis was used as class-defining variables in the model (Table S2–S6). Details of data handling and procedures used for model development can be found in the online supplement. Ventilator-free days (VFDs; censored at day 28) and in-hospital mortality were the main outcome variables and excluded from the modelling.

In both cohorts, differences in chronic health conditions were analysed that had a cohort prevalence of ≥10%, were associated with increased risk for developing ARDS or were known to modulate inflammation. Additionally, substance abuse data were also compared between the subphenotypes. In EARLI, data for several novel plasma biomarkers were measured that are known to be implicated in the pathogenesis of ARDS in human or animal models and were used to further characterize the subphenotypes. These new biomarkers were selected prospectively to study (A) the extracellular matrix: matrix metalloproteinases (MMP): MMP-8 (collagenase) and MMP-9 (gelatinase);[11–13] (B) chemokines: macrophage inflammatory protein (MIP)1-α and chemokine ligand (CCL)-8;[14] (C) endothelial injury: von Willebrand factor (vWF), angiopoietin (Ang)-2 and vascular endothelial growth factor (VEGF);[15, 16] (D) lung epithelial injury: surfactant protein (SP)-D and receptor for advanced glycation end-products (RAGE).[17]

### Assay Procedure

Biomarkers were measured based on our prior studies of ARDS subphenotypes. For each cohort, biomarkers were measured at their respective sites and in some instances using different assays. All biomarkers were quantified using either single- or multiplex enzyme-linked immunosorbent assays. Details of the biomarkers used for LCA have been described previously.[7, 17] A summary of the assay procedures can be found in the online supplement.

### Parsimonious Classifier Models

Parsimonious classifier models previously derived and validated in RCTs can be used to classify LCA-derived ARDS subphenotypes with high accuracy.[18] In order to determine concordance of the subphenotypes identified in the current cohorts with those identified in prior RCT cohorts, the accuracy of two of these logistic regression classifier models, the 3-variable model (interleukin-8, serum bicarbonate and protein C) and the 4-variable model (addition of vasopressor-use to the 3-variable model), was tested in all the described cohorts. Receiver-operating characteristic curves were constructed for the models and the area under the curve (AUC) was used to evaluate model performance with LCA-derived subphenotype serving as the gold standard. Details of the parsimonious model development can be found in the supplement.

Differences between the classes were tested using either the Student’s t-test, Wilcoxon rank sum test, or Chi-square test, depending on the type and distribution of the data. Kaplan-Meier survival curves, censored at day 60, were plotted to compare survival across subphenotypes. LCA was performed using Mplus (version 8.1). All other analyses were performed using R-software on RStudio version 1.0.143.

## Results

Description of the population characteristics and outcomes in VALID and EARLI are presented in Table 1. There were notable differences between the cohorts. Compared to EARLI, patients in VALID were younger and predominantly white. 36% were current smokers in VALID compared to 15% in EARLI. In VALID, trauma was the risk factor for ARDS in 28% of the patients, whereas in EARLI, trauma patients were excluded by design (Table S1). At enrollment, vasopressor-use was higher in EARLI (63% vs 43%), but invasive ventilation was lower (68% vs 80%). In-hospital mortality was higher in EARLI compared to VALID (41% vs 27%).

### 2-Class LCA Model Best Fit the VALID Cohort

Based on the fit statistics (Table 2), the 2-class model best fit the VALID AECC cohort. Bayesian Information Criteria (BIC) decreased the greatest between a 1-class and 2-class model (Table 2). The VLMR test was significant for the 2-class model (p=0.001). Although further increases in the number of classes led to improved model fit, the VLMR test were not significant for these models. Taken together, the 2-class model was adjudicated as the best-fitting model. In the 2-class model, 457 (73%) of the patients were classified as subphenotype 1 and 167 (27%) of the patients were classified to subphenotype 2. The mean probability for class assignment was 0.98 for subphenotype 1 and 0.93 for subphenotype 2, indicating good separation.

In VALID, given the high proportion of trauma patients and that 94% of these patients were in Class 1 (Table S2), a further LCA was performed excluding patients with trauma-associated ARDS. Mortality in the non-trauma cohort was higher than the overall VALID mortality (33% vs 27%). Comparison of the fit statistics once again indicated that the 2-class model best fit the cohort (p=0.0129; Table S7). There were 315 (70%) patients in subphenotype 1, and 137 (30%) patients in subphenotype 2, and the probabilities for class membership were 0.96 and 0.95 respectively.

Using the same procedures described above, in a secondary analysis of VALID that only included patients that met the Berlin ARDS definition, the 2-class model once again best fit this cohort with similar proportions of patients in the two subphenotypes as in the cohort that met the AECC definition (Table S8).

### 2-Class Model Best Fit the EARLI Cohort

In EARLI patients who met the AECC criteria, the 2-class model also best fit the population. BIC decrease was greatest between the 1-class and 2-class model and the latter was the only model with a significant VLMR test (p<0.0001, Table 2). For the 2-class model, 211 (63%) of the patients were in subphenotype 1, and 124 (37%) of the patients were in subphenotype 2, with a mean probability of class membership of 0.96 and 0.95 respectively. Similar findings were observed with LCA of a subset of patients that only met the Berlin definition of ARDS (Table S9).

### Distinct characteristics and outcomes were observed in ARDS Subphenotypes

Due to the consistent findings in both cohorts and their subsets, the 2-class model was retained for all downstream analysis. Further, the subset VALID that excluded trauma-associated ARDS was also included as a third cohort for the primary analysis for the remainder of the manuscript.

**Figure 2.**
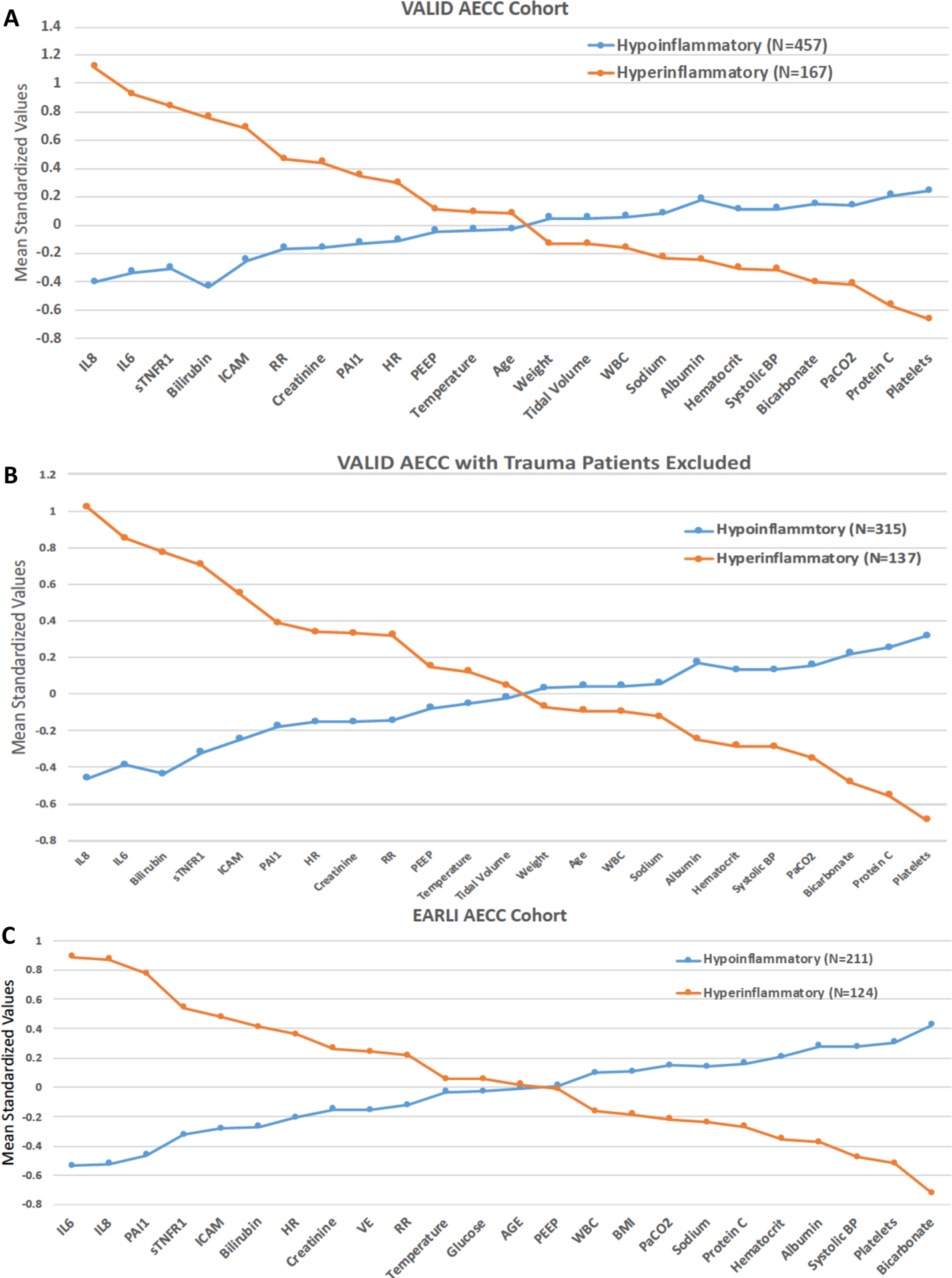
Standardized values for continuous class-defining variables used in the latent class models. The variables are sorted from left to right in descending order for the highest values in the Hyperinflammatory subphenotype. Standardized values were calculated by assigning the mean of the variables as 0 and standard deviation as 1. 2A VALID Cohort (AECC Definition). 2B VALID Cohort (AECC Definition with Trauma patients excluded). 2C VALID Cohort (AECC Definition). BMI: body mass index, SBP: systolic blood pressure, ICAM-1: intercellular adhesion molecule-1, IL-6: interleukin 6, IL-8: interleukin 8, PAI-1: plasminogen activator inhibitor-1, PEEP: positive end-expiratory pressure, sTNFr1: tumor necrosis factor receptor-1, VE: minute ventilation, VT: tidal volume, WBC: white blood cell count, RR = Respiratory Rate, HR = Heart Rate.

For the primary cohorts, Subphenotype 2 was characterized by elevated plasma levels of pro-inflammatory markers such as IL-8, IL-6 and sTNFR-1. Subphenotype 2 was also characterized by lower levels of bicarbonate, platelets and protein C (Figure 2A–C). The characteristics of subphenotypes 1 and 2 (Table S2–3, S6) were in keeping with the previously identified Hypoinflammatory and Hyperinflammatory subphenotypes respectively (referred as thus herein). Vasopressor-use was significantly more prevalent in the Hyperinflammatory subphenotype compared to the Hypoinflammatory subphenotype (VALID 62% vs 36%, p<0.0001; EARLI 94% vs 45%, p<0.0001; Figure S2). Importantly, bilirubin was also higher in the Hyperinflammatory subphenotype and compared to prior RCT LCA studies, featured more prominently as a class-defining variable in VALID.

In VALID, there was no significant difference between the subphenotypes in either the hypoxia categories (p=0.60) or incidence of invasive ventilation (p=0.94) (Figures S3A–S3B). In EARLI, the difference in hypoxia categories and frequency of invasive ventilation reached statistical significance, although, the absolute difference in proportions were between 10–20% (Figure S3C). In VALID (after the exclusion of trauma patients) and EARLI, sepsis was the most common risk factor for ARDS in the Hyperinflammatory subphenotype, and pneumonia was the most common in the Hypoinflammatory subphenotype (Tables S3 and S6).

Across all cohorts, the APACHE scores were significantly higher in the Hyperinflammatory subphenotype (Table S10). Clinical outcomes were also significantly worse in the Hyperinflammatory subphenotype including fewer VFDs and higher in-hospital mortality which was between 50% – 60% across all three primary cohorts (Table 3). Survival in Hyperinflammatory phenotype was significantly worse than the Hypoinflammatory phenotype (p < 0.0001 both cohorts; Figure S4).

In both VALID and EARLI, the observed differences in the clinical characteristics and clinical outcomes of subphenotypes were similar in secondary analyses of Berlin criteria for ARDS (Tables S4, S5 and S11).

### Co-morbidities differed between the Subphenotypes

**Figure 3.**
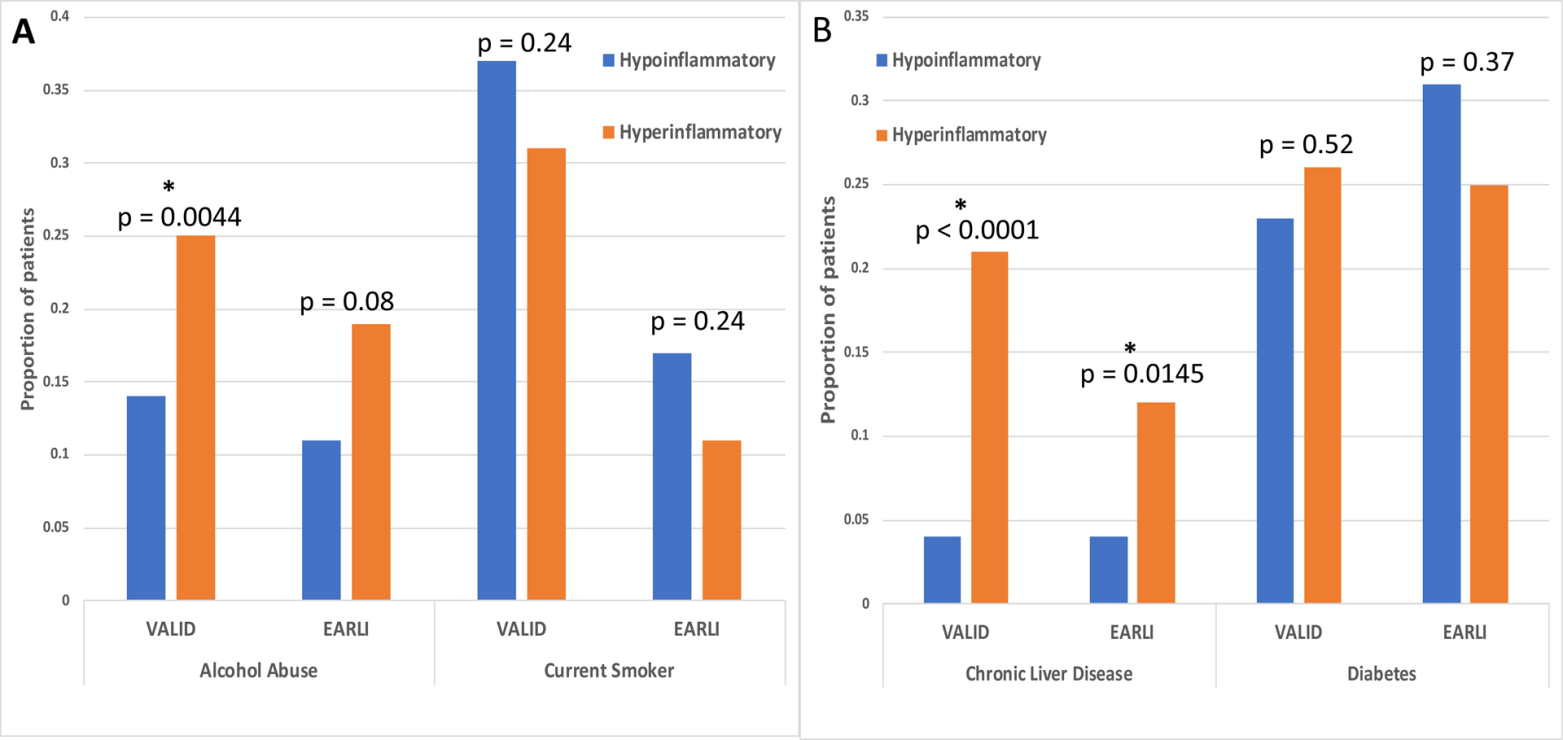
Comparison of substance abuse and selected co-morbidities between subphenotypes. 3A: Alcohol abuse and current smoking status. 3B: Chronic liver disease and diabetes. P-values are derived using Chi-squared test. * Denotes statistically significant value.

Both VALID and EARLI had a greater depth of clinical information available compared to prior RCT LCAs. These novel data were excluded from the LCA models and were instead used to characterize the ARDS subphenotypes. History of alcohol abuse was more prevalent in the Hyperinflammatory compared to Hypoinflammatory subphenotype (VALID p=0.004; EARLI p=0.078; Figure 3A). Conversely, there were more current smokers in the Hypoinflammatory subphenotype, though this difference was not statistically significant in either cohort (VALID p=0.24; EARLI p=0.24; Figure 3A).

A history of chronic liver disease / cirrhosis was observed in a significantly greater proportion of patients in the Hyperinflammatory compared to the Hypoinflammatory subphenotype (VALID p<0.0001; EARLI p=0.0145; Figure 3B). There were no significant differences in the prevalence of diabetes (types I and II) between the subphenotypes in either cohort (Figure 3B). In EARLI, COPD was more prevalent in the Hypoinflammatory compared to the Hyperinflammatory subphenotype (26% vs 12%; p=0.005). This trend was similar in VALID but not statistically significant (Hypoinflammatory 16% vs Hyperinflammatory 11%; p=0.21). The prevalence of a history of solid tumors was similar between the two classes (VALID: Hypoinflammatory 9% vs Hypoinflammatory 12%, p=0.25; EARLI: Hypoinflammatory 19% vs Hyperinflammatory 25%, p=0.99).

In both cohorts, neutropenia (defined as white cell count < 1.5×10^3^/μL) was more prevalent in the Hyperinflammatory subphenotype (VALID: 14% vs 0.8%, p<0.0001; EARLI: 9% vs 1%, p=0.0027). Chronic corticosteroid therapy use was similar between the subphenotypes (VALID: Hyperinflammatory 19% vs Hypoinflammatory 14%, p=0.1768; EARLI: Hyperinflammatory 8% vs Hypoinflammatory 8%, p=0.29).

In a subset of patients, data pertinent to cardiac function and myocardial injury were available. In VALID, left ventricular ejection fraction on transthoracic echocardiography during hospitalization, and in EARLI, baseline Troponin-I and brain-natriuretic protein (BNP), were available. Distribution of values in all three metrics were similar between the two subphenotypes (see Table S12).

### Parsimonious classifier models performed with high accuracy in both cohorts

**Figure 4.**
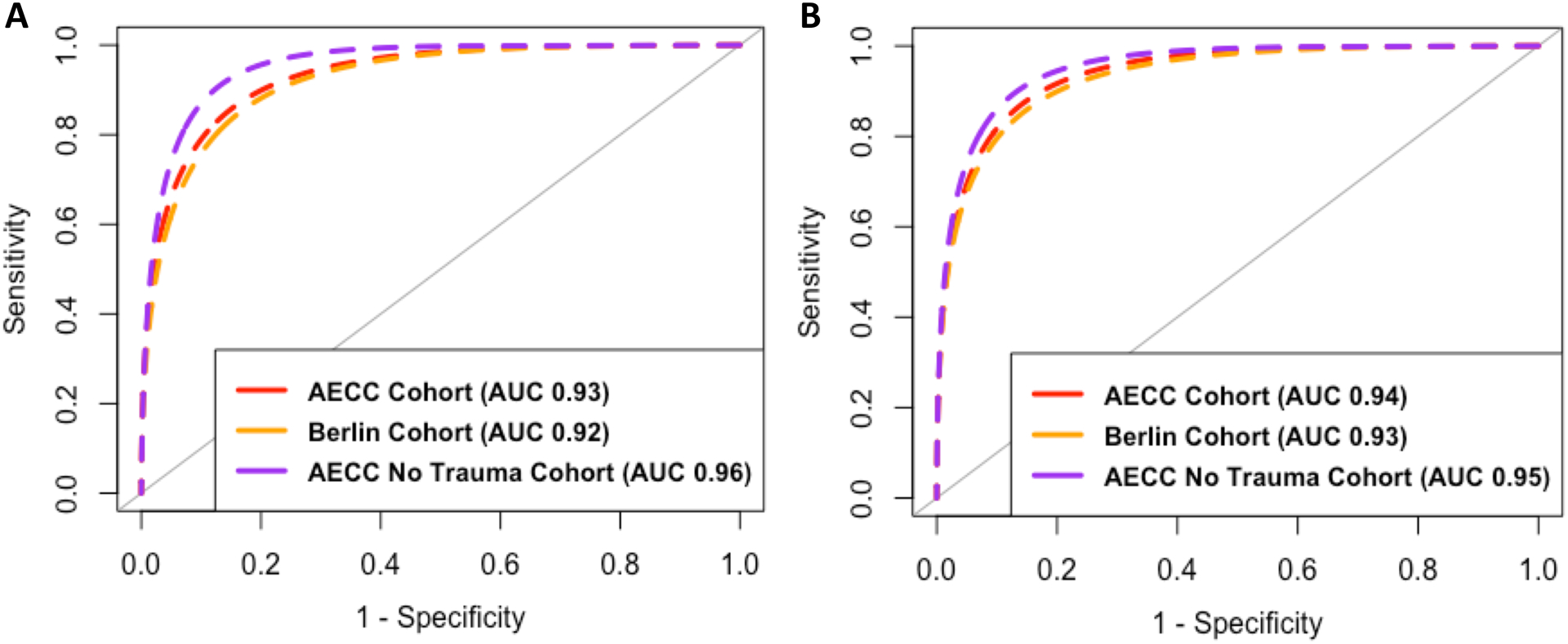
Receiver operating characteristic (ROC) curves for the parsimonious classifier models in the three VALID cohorts: AECC (n = 624), No trauma (n = 452) and Berlin (n = 500) Cohorts. 4A: ROC curves for the 3-variable model (interleukin-8, serum bicarbonate and protein c). 4B: ROC curves for the 4-variable model (interleukin-8, serum bicarbonate, protein c and vasopressor-use). AUC: Area under the curve.

In VALID, the previously developed and validated 3-variable (AUC 0.93 95% CI: 0.91–0.95) and 4-variable (AUC 0.94 95% CI: 0.92–0.96) parsimonious classifier models had high performance indices in classifying LCA subphenotypes (Figure 4). The classification accuracy using a cut-off of 0.5 for classification was 86% in the 3-variable model and 88% for the 4-variable model. The 3-variable model had higher performance metrics after the trauma patients were excluded (AUC 0.96 95% CI: 0.94–0.97). Both models performed similarly in all other subsets of VALID (Figure 4A and Figure 4B). Likewise, in EARLI, the 3-variable and 4-variable models had AUCs ≥ 0.93 in both the AECC and Berlin cohorts (Figures S5A and B).

### Differences in Novel Protein Biomarkers were observed between subphenotypes in EARLI

**Figure 5.**
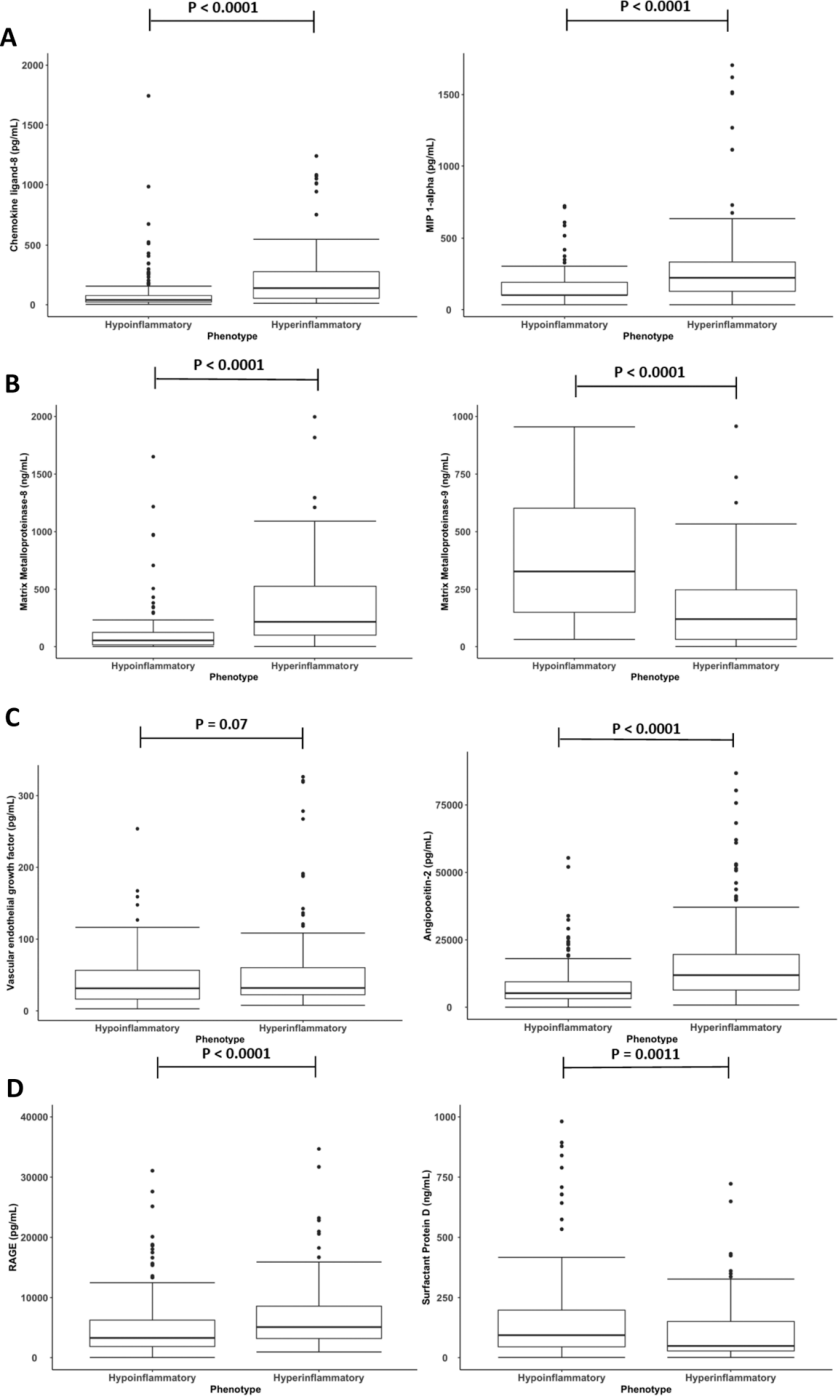
Comparison of differences in novel plasma biomarkers between subphenotypes. 5A: Matrix metalloproteinases. 5B: Chemokines). 5C: Markers of endothelial injury. 5D: Markers of epithelial injury. MIP = Macrophage Inflammatory Protein; RAGE = Receptor for Advanced Glycation Endproducts. Protein P-values are derived using Wilcoxon-rank test.

The sample availability for the novel biomarkers and their distribution in each subphenotype are summarized in Table S13 and their correlations with each other are presented in Figure S6. MMP-8 was significantly higher in Hyperinflammatory subphenotype, whereas, MMP-9 was significantly lower (Figure 5A). The chemokines CCL-8 and MIP-1α were significantly higher in the Hyperinflammatory subphenotype (Figure 5B). Angiopoietin-2 and vWF were also significantly higher in the Hyperinflammatory subphenotype, whereas, no significant difference in VEGF levels were observed between the subphenotypes (Figure 5C). RAGE was significantly higher in the Hyperinflammatory subphenotype. Conversely, SP-D was significantly higher in the Hypoinflammatory subphenotype (Figure 5D).

## Discussion

Using LCA, two ARDS subphenotypes were identified in two distinct observational cohorts from two university medical centers, one located in San Francisco, California and the other in Nashville, Tennessee. The geographically-driven demographic diversity observed between the two cohorts make the consistent findings of the two subphenotypes more remarkable. The clinical characteristics, outcomes and biological profile of the two subphenotypes were similar to those previously identified in five RCTs.[2–5] Additionally, parsimonious classifier models developed and validated in RCT populations identified LCA-derived subphenotypes in these observational cohorts with high accuracy, indicating concordance with previously described ARDS subphenotypes. These findings suggest that the Hyperinflammatory and Hypoinflammatory ARDS subphenotypes are generalizable to unselected populations of ARDS.

Although considerable concordance was observed between subphenotypes identified in this study and previous RCTs, there are some interesting differences that may partly be explained by the differences in these populations. For example, patients with severe liver dysfunction are excluded from most ARDS RCTs and have been poorly studied in these subphenotypes. Interestingly, inclusion of such patients in the present study may have resulted in bilirubin being a more important class-defining variable compared to prior RCT LCA studies. Further, end-stage liver disease (ESLD) is known to be a barrier to ARDS resolution[19] and impairs inactivation of inflammatory mediators during sepsis leading to sustained elevation in IL-6 and IL-8.[20, 21] Both these factors may explain the higher prevalence of ESLD in the Hyperinflammatory subphenotype.

Gadre and colleagues observed that in patients admitted to the ICU with acute respiratory failure requiring mechanical ventilation, those with COPD exacerbations presented with lower APACHE III scores and had more favourable clinical outcomes, including lower mortality, compared to other leading causes of acute respiratory failure.[22] These patterns are congruous with the higher prevalence of COPD observed in the Hypoinflammatory subphenotype in this study.

Neutropenic patients were predominantly confined to the Hyperinflammatory subphenotype. These findings suggest that the source of inflammatory biomarkers in plasma that define this subphenotype is likely not limited to neutrophils, even though they normally constitute the most abundant circulating leukocyte. Further, the findings of elevated inflammatory biomarkers in neutropenia in this subphenotype mirror the sustained elevated serum levels of IL-6 and IL-8 that are frequently observed in bacteraemic patients with neutropenic sepsis.[23, 24] In a population of patients with neutropenic sepsis requiring ICU admission, Reilly and colleagues also observed higher levels of circulating pro-inflammatory cytokines including granulocyte colony-stimulating factor (G-CSF) compared to non-neutropenic sepsis patients in the ICU.[25] They also observed that exogenous G-CSF was associated with increased levels of plasma IL-8 and IL-6. The prevalence of bacteraemia and use of G-CSF were not uniformly available in both cohorts, and drivers of the hyperinflammatory state in neutropenic patients warrants further evaluation.

In EARLI, data were available for a novel panel of plasma biomarkers. Most biomarkers were divergent in the subphenotypes despite not being used as class-defining variables in the LCA. MMP-8 and MMP-9 are both known be elevated in early critical illness including ARDS and have been implicated in its pathogenesis through the destruction of components of the extracellular matrix.[13, 26, 27] In EARLI, both these markers were higher than the normal range described by the manufacturer of the assay (measured in healthy volunteers). When stratified by subphenotypes, however, their values became divergent. MMP-8 levels were higher in the Hyperinflammatory, whereas, MMP-9 levels were higher in the Hypoinflammatory subphenotype. The interaction of MMPs in relation to acute inflammation is complex and depending on the phase of illness, they may either contribute to injury or promote repair.[28] In a series of experimental murine models of ventilator-induced lung injury, Albeiceta and colleagues demonstrated that MMP-8 deficiency was protective, whereas, MMP-9 deficiency potentiated injury.[29, 30] Further, Zinter and colleagues, using LCA based on plasma MMPs, identified two distinct subphenotypes in pediatric ARDS.[31] The second subphenotype, which was driven largely by lower levels of MMP-9, was associated with worse clinical outcomes and similar to the Hyperinflammatory subphenotype in the presented study.

Another interesting finding was observed in the distribution of markers of lung epithelial injury. SP-D levels were significantly higher in the Hypoinflammatory subphenotype. In contrast, RAGE levels were significantly higher in the Hyperinflammatory subphenotype. Elevated plasma RAGE, in part, reflects injury to alveolar epithelial cells that cover more than 90% of the normal barrier, and is been associated with reduced alveolar fluid clearance in experimental models and in patients with ARDS.[32] However, RAGE has also been implicated as an integral molecule in the innate immune response in septic shock and elevated plasma levels are associated with adverse clinical outcomes.[33, 34] Lung epithelia aside, other epithelial sources such as the kidneys, may also contribute to the elevated circulating levels of RAGE, particularly in the setting of systemic inflammation as seen in the Hyperinflammatory subphenotype. The correlation between creatinine and RAGE in EARLI (r=0.48) would reinforce the theory of an association between RAGE and renal impairment. Whether this association is as a consequence of increased injury or impaired clearance cannot be answered using the current data alone and warrants further investigation.

It is also noteworthy that SP-D, a specific marker of alveolar epithelial injury, was higher in the Hypoinflammatory phenotypes, whereas markers of endothelial injury were higher in the Hyperinflammatory phenotype. These findings are consistent with pneumonia being the predominant risk factor of the Hypoinflammatory phenotype and the greater proportion of sepsis-ARDS in the Hyperinflammatory phenotype. Prior studies have shown a similar pattern of elevated biomarkers of epithelial and endothelial in direct and indirect ARDS respectively.[17]

Considerable differences between the two studied cohorts were observed, not least that mortality, rates of mechanical ventilation, and vasopressor use were higher in EARLI compared to VALID. Differences in demographics, socioeconomic status and chronic health between the populations served by the hospitals from which the cohorts were drawn have likely resulted in observed diversity. Patients presenting to VALID were younger than in EARLI, and in part, this may explain their more favourable outcomes. Further, VALID was conducted at a Level I trauma centre, a large proportion of patients had trauma-associated ARDS (28%) with a lower mortality (9%) compared to the cohort at large. In contrast, trauma-associated ARDS was excluded from EARLI.

Trauma-associated ARDS differs biologically and clinically from other causes of ARDS, including a lower age-adjusted mortality.[35] In prior LCA studies in RCTs, trauma constituted only 7% of the combined population. Crucially, most of these patients were classified as the Hypoinflammatory subphenotype suggesting a much lower burden of inflammation. Further, once these patients were removed from the analysis, the proportion of patients per subphenotype was more in line with prior ARDS subphenotypes and also led to improved parsimonious model performance. It may be that the distinct characteristics of trauma-associated ARDS mandates that these patients be studied separately when performing clustering analyses.

Other investigators have used other approaches to identify clusters in observational cohorts of ARDS [36] and have similarly identified two subphenotypes with markers of inflammation being the central differentiating factor. Assumptions that these subphenotypes are the same as those identified in our work are not valid because the methods and variables used were different and this warrants further investigation. Regardless, taken together these studies indicate that inflammation, as measured by a panel of inflammatory biomarkers, is a robust discriminator of patients with ARDS. In order to progress and apply these subphenotypes clinically, certain conditions need to be met. These include a phenotyping approach that is consistent, reproducible and identifiable at the bedside such that it could potentially make a difference in the clinical workflow. The presented study, in conjunction with our prior work, establishes the consistency, reproducibility and generalizability of the hyper- and hypoinflammatory phenotypes. Further, in a recent study we have demonstrated the feasibility of using the parsimonious model prospectively to classify patients in real-time using a point-of-care assay.[37]

There are several limitations in this study. First, the biological samples were collected at different time-points in the two cohorts. It was not possible to ascertain whether the differences between cohorts in the proportion of patients per subphenotype was due to biological differences between the populations or due to the longitudinal kinetics of ARDS. Second, inter-assay variability may also have resulted in the observed differences in biomarkers between the cohorts. The consistent findings of the two subphenotypes despite these differences, alongside the performance of the parsimonious models, arguably enhances the generalizability of ARDS subphenotypes. In EARLI, consent was waived in patients who died prior to it being obtained, and therefore, these patients may have been included at higher rates. This approach may have introduced bias by including patients with worse outcomes. Both cohorts analysed in this study were from the United States of America, and the generalizability to other healthcare systems needs further evaluation. Finally, patients were not recruited continuously to either study, and biases inherent to convenience sampling may be applicable to this study.

## Summary

In two independent observational cohorts of ARDS, the hypo- and hyperinflammatory subphenotypes were identified indicating their generalizability to non-trauma populations. Importantly, these findings suggest that future subphenotype-based clinical trials may also be applicable to such populations. Further, we observed that the hyperinflammatory subphenotype has distinct features characterized by elevated markers of endothelial injury and lower levels of MMP-9 compared to the hypoinflammatory subphenotype. This study addresses several key knowledge gaps in our understanding of LCA-derived ARDS subphenotypes and represents an important step forward in their clinical application.

## Supplementary Material

FigureE2

FigureE3

FigureE4

FigureE5

FigureE1

Supplement Text

FigureE6

## Figures and Tables

**Table 1.** Population Characteristics of the VALID and EARLI cohorts for all patients that met the AECC criteria for ARDS. Data are presented as n (%), mean (± Standard deviation), or median (interquartile range).

	VALID (n = 624)	EARLI (n = 335)
**Age (Years)**	53 (± 17)	66 (± 17)
**Sex: Female**	261 (42%)	147 (44%)
**Race: White**	544 (87%)	165 (49%)
**Weight (kg)**	84 (± 27)	76 (± 30)
**Alcohol abuse**	107 (17%)	46 (14%)
**Current smoker**	223 (36%)	49 (15%)
**Diabetes**	151 (24%)	79 (29%)
**Chronic liver disease**	51 (8%)	24 (7%)
**Temperature (°C)**	38 (± 1.1)	38 (± 1.4)
**Heart rate (beats.min** ^**−1**^ **)**	122 (± 21)	126 (± 27)
**Systolic blood pressure (mmHg)**	87 (± 16)	86 (± 21)
**Hypoxia Category**		
**Mild**	155 (25%)	84 (25%)
**Moderate**	263 (42%)	126 (38%)
**Severe**	206 (33%)	125 (37%)
**PaCO**_**2**_ **(mmHg)**	49 (± 14)	43 (± 16)
**Tidal Volume (mL)**	441 (± 77)	432 (± 79)
**Respiratory rate (breaths.min** ^**−1**^ **)**	29 (24 – 35)	35 (30 – 40)
**Haematocrit (%)**	29.6 (± 7)	30.4 (± 7)
**White cell count (10** ^**3**^ **/μL)**	16.2 (± 12)	15.0 (± 13)
**Platelets (10** ^**3**^ **/μL)**	167 (± 113)	169 (± 105)
**Sodium (mmol/L)**	139 (± 5)	135 (± 6)
**Creatinine (mg/dL)**	1.8 (± 1.5)	2.1 (± 2.2)
**Bicarbonate (mmol/L)**	21 (± 5)	21 (± 6)
**Albumin (g/dL)**	2.6 (± 0.6)	2.5 (± 0.7)
**Bilirubin (mg/dL)**	1.1 (0.7 – 2.1)	1.0 (0.7 – 1.5)
**Interleukin-6 (pg/mL)**	55 (21 – 209)	191 (51 – 2471)
**Interleukin-8 (pg/mL)**	16 (7 – 60)	32 (12 – 210)
**Soluble tumor-necrosis factor receptor-1 (pg/mL)**	2384 (1467 – 4253)	4053 (2246 – 10116)
**Intercellular adhesion molecule 1 (ng/mL)**	413 (271 – 677)	667 (347 – 1241)
**Protein C (% control)**	66 (± 39)	90 (± 68)
**Plasminogen activator inhibitor 1 (ng/mL)**	19.6 (9 – 37)	8 (3 – 30)
**Primary ARDS risk factor**		
**Pneumonia**	156 (25%)	145 (43%)
**Sepsis**	148 (24%)	118 (35%)
**Aspiration**	108 (17%)	51 (15%)
**Trauma**	172 (28%)	–
**Other**	40 (6%)	21 (6%)
**Vasopressor use at baseline**	268 (43%)	212 (63%)
**Invasive Ventilation at baseline**	500 (80%)	225 (68%)
**APACHE II Score**	28 (± 8)	30 (± 10)
**Ventilator Free Days (censored at day 28)**	18 (4 – 24)	21 (0 – 26)
**In-hospital Mortality** *	167 (27%)	137 (41%)

* Followed up to day 60 in EARLI and to discharge in VALID

**Table 2:** Fit statistics for the latent class analysis models applied to the primary cohorts: Patients that met AECC definition for ARDS in the VALID cohort and in the EARLI cohort.

	Classes	BIC	VLMR p	N_1_	N_2_	N_3_	N_4_	Entropy
**VALID AECC Cohort**	1	43315	–	624	–	–	–	–
	2	42454	0.0010	457	167	–	–	0.87
	3	42213	0.74	303	187	134	–	0.90
	4	42126	0.36	231	190	127	76	0.89
	Classes	BIC	VLMR p	N_1_	N_2_	N_3_	N_4_	Entropy
**EARLI AECC Cohort**	1	24235	–	335	–	–	–	–
	2	23650	< 0.0001	211	124	–	–	0.86
	3	23550	0.18	166	117	52	–	0.87
	4	23535	0.35	164	116	52	3	0.90

AECC = American-European Consensus Conference. BIC = Bayesian Information Criteria. VLMR = Vuong-Lo-Mendel-Rubin test. N represents the number of observations in each class.

**Table 3:** Clinical outcomes in the primary cohorts stratified by ARDS subphenotypes.

	Ventilator-Free Days			In-hospital mortality*		
	Hypo-inflammatory	Hyper-inflammatory	P-value	Hypo-inflammatory	Hyper-inflammatory	P-value
**VALID AECC**	20 (11 – 25)	5 (0 – 20)	< 0.0001	80/457 (18%)	87/167 (52%)	< 0.0001
**VALID AECC (NO TRAUMA)**	21 (8 – 25)	4 (0 – 18)	< 0.0001	71/315 (23%)	80/137 (58%)	< 0.0001
**EARLI AECC**	24 (0 – 28)	0 (0 – 23)	< 0.0001	62/211 (29%)	75/124 (60%)	< 0.0001

P- value represent Wilcoxon rank sum test for ventilator free days and chi-squared test for in hospital mortality. AECC = American-European Consensus Conference.

* Followed up to day 60 in EARLI and to discharge in VALID

## Key Messages

### Key question:

Are the previously described phenotypes of ARDS identified in randomised controlled trials, generalizable to observational cohorts of ARDS?

### Bottom line:

The hypoinflammatory and hyperinflammatory subphenotypes of ARDS that had previously been identified in 5 RCTs are now generalizable to observational cohorts of ARDS, and the biological and prognostic information associated with the two subphenotypes are applicable to unselected ARDS populations.

### Read on:

To get a better understanding and mapping of the clinical and biological characteristics of the two phenotypes.
