# Supplementary figures and images for: Latent Class Analysis-Derived Subphenotypes are Generalizable to Observational Cohorts of Acute Respiratory Distress Syndrome: A Prospective Study
Source: Thorax. Author manuscript; Available in PMC 2023 Jan 1. (PMC8688287; doi:10.1136/thoraxjnl-2021-217158)

# Vasopressor Use

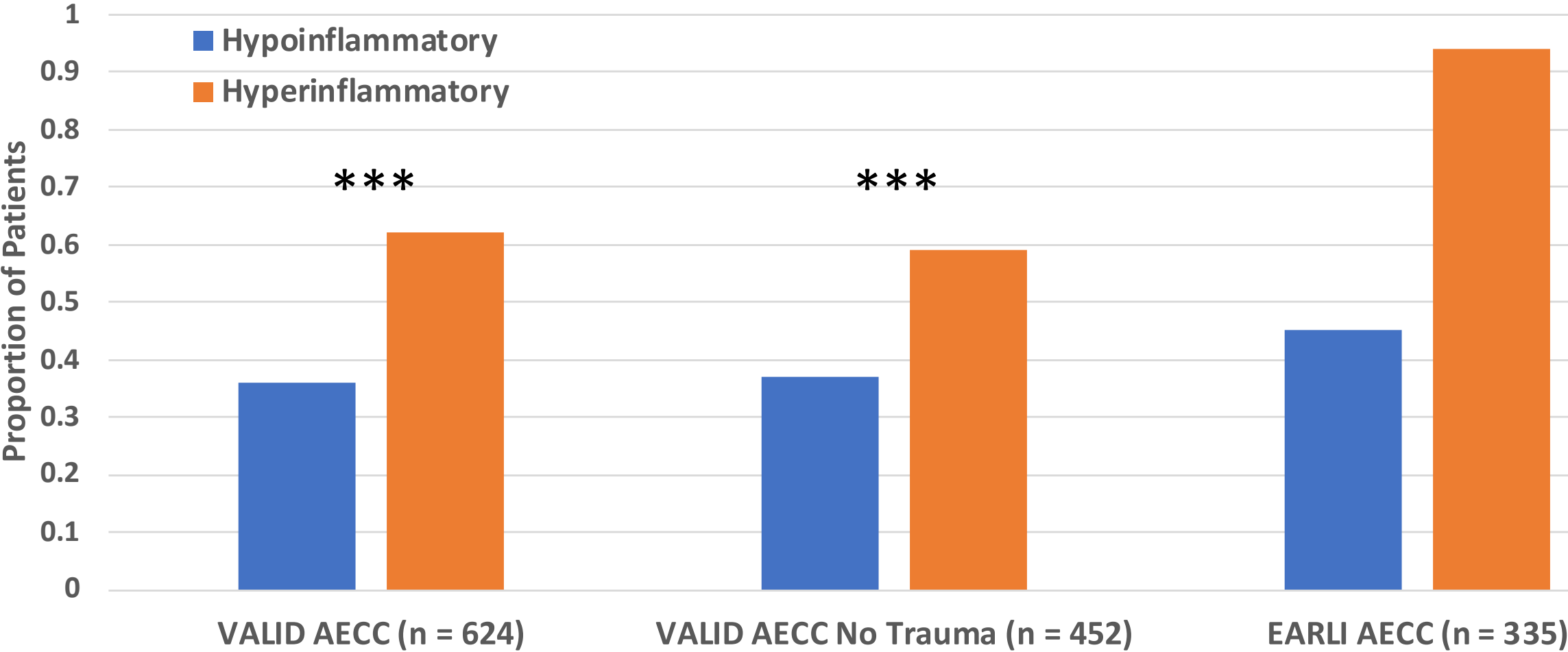

Supplement: FigureE2 [file NIHMS1753178-supplement-FigureE2.pdf]

**A****VALID AECC**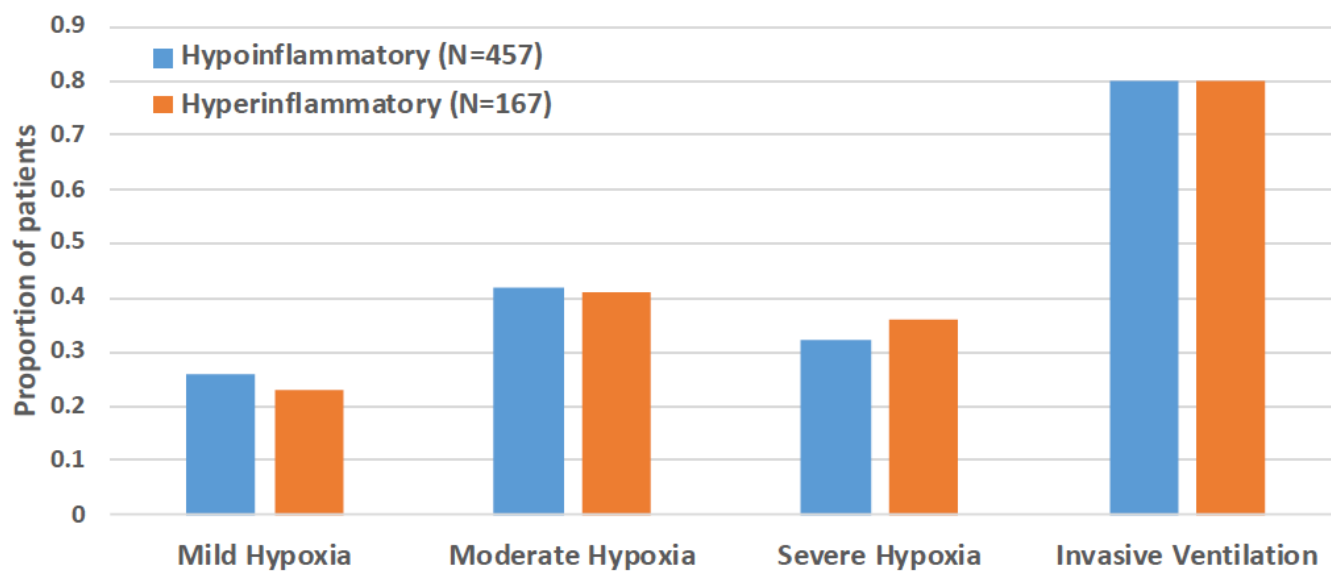**B****VALID AECC (No trauma)**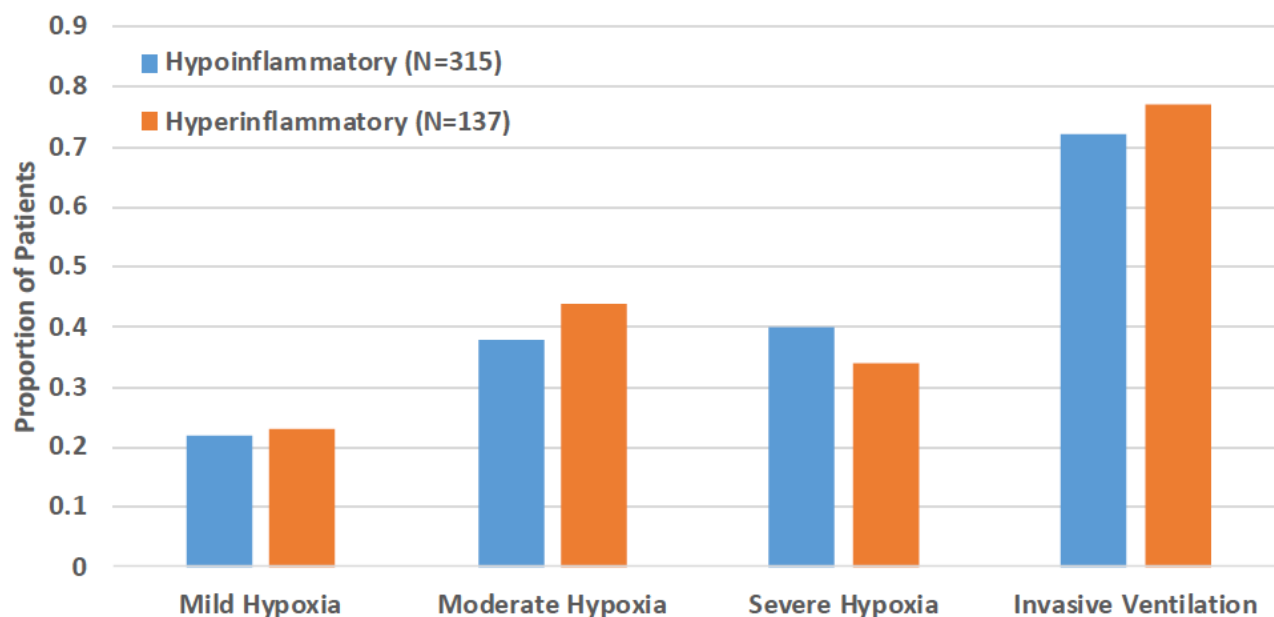**C****EARLI AECC**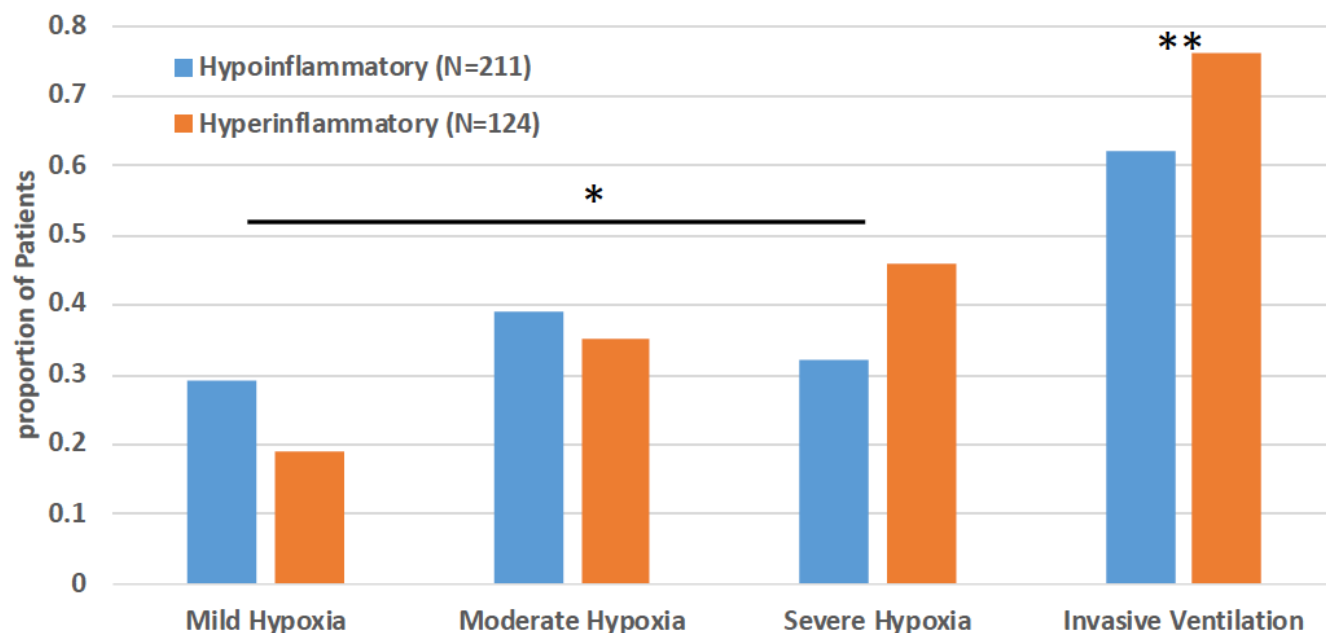

Supplement: FigureE3 [file NIHMS1753178-supplement-FigureE3.pdf]

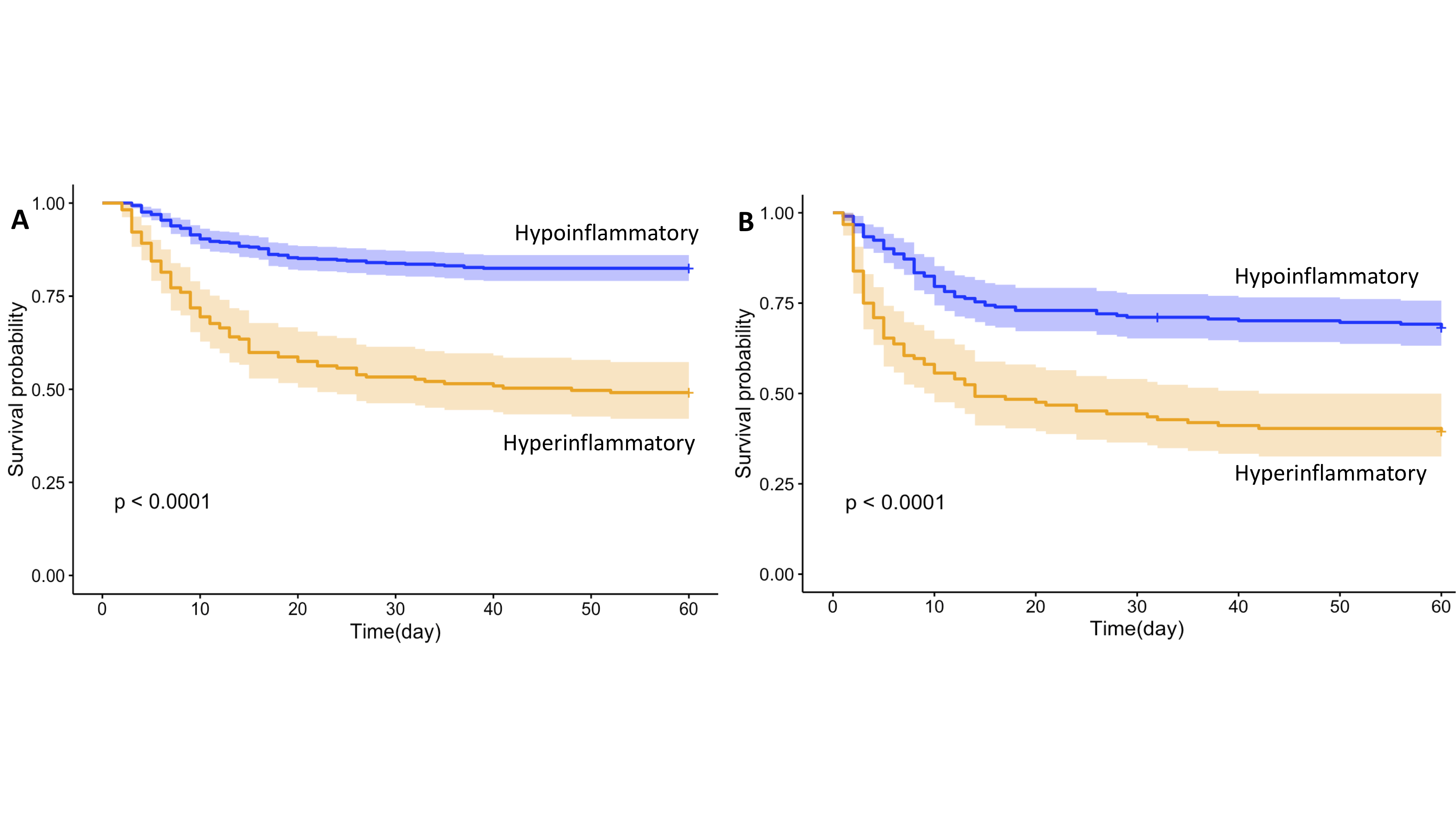

Supplement: FigureE4 [file NIHMS1753178-supplement-FigureE4.png]

**A**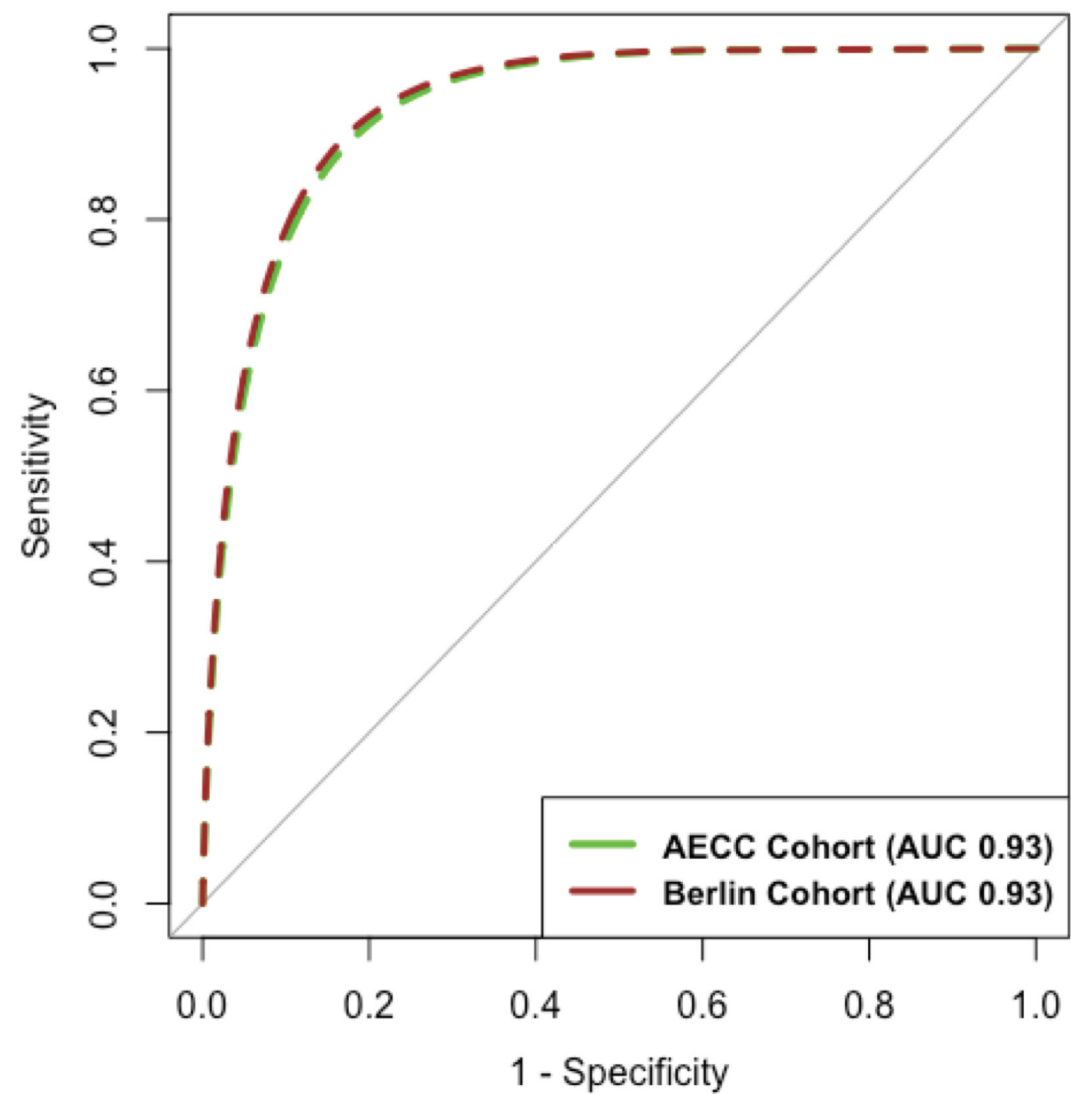**B**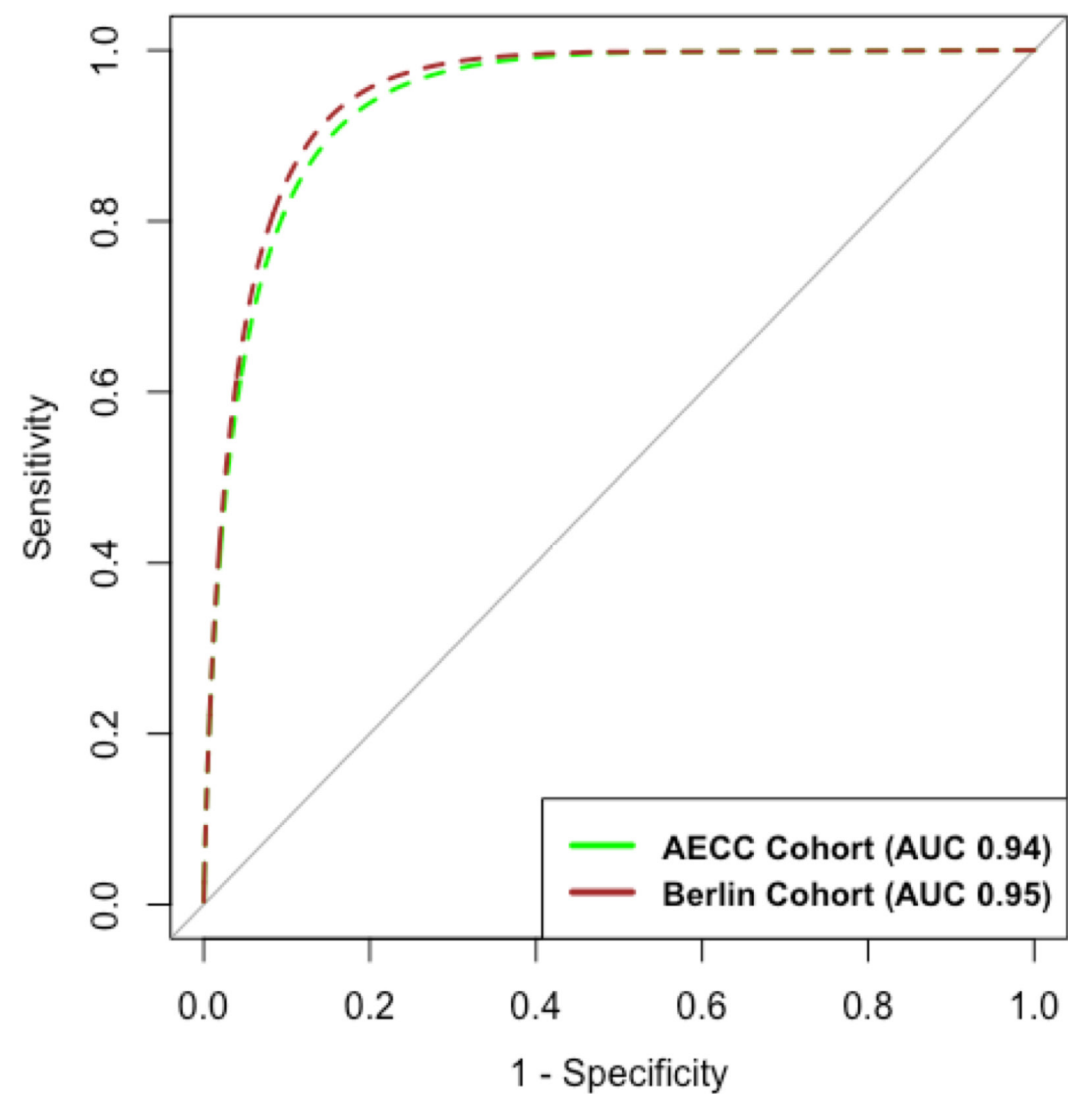

Supplement: FigureE5 [file NIHMS1753178-supplement-FigureE5.pdf]

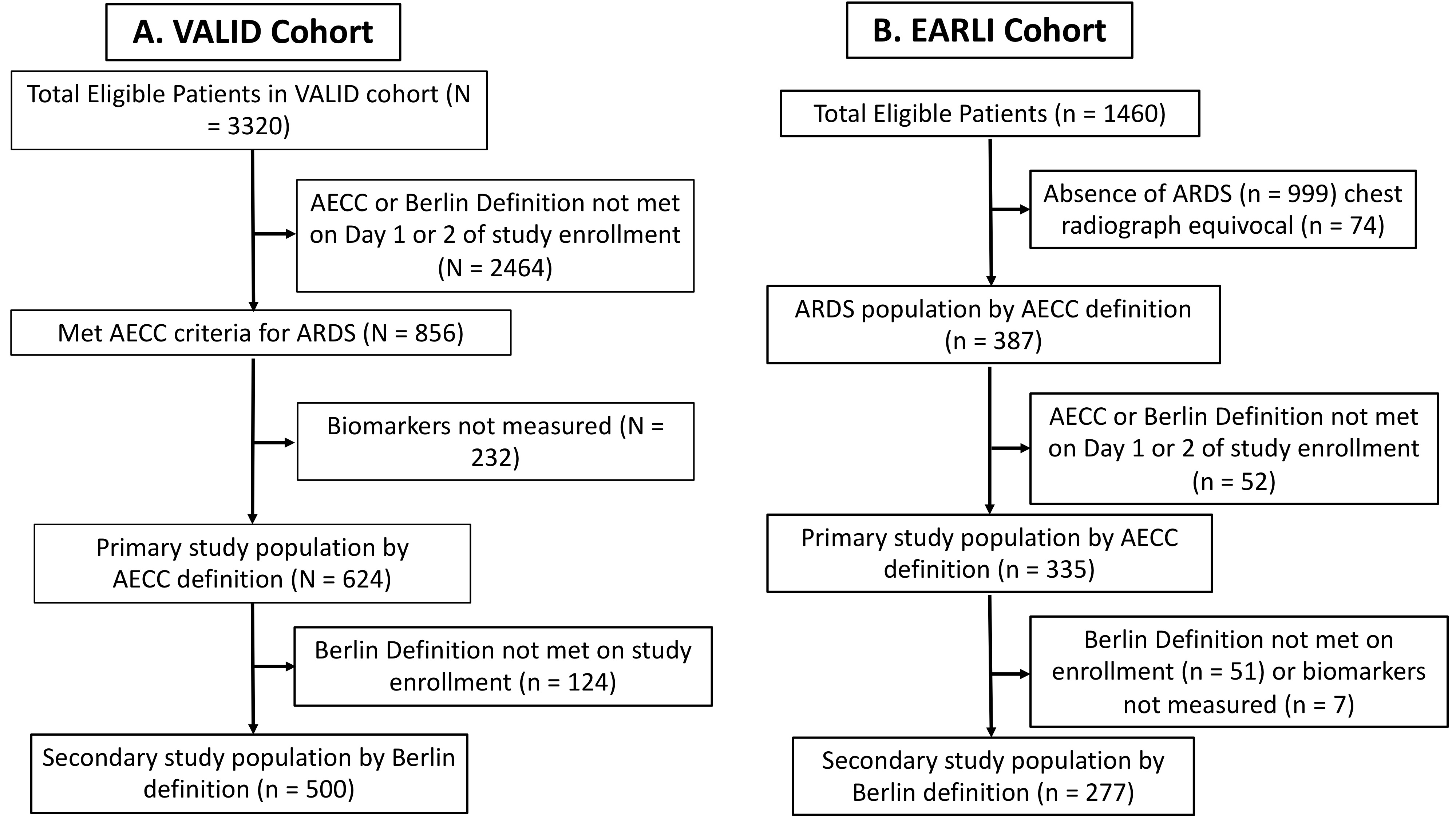

Supplement: FigureE1 [file NIHMS1753178-supplement-FigureE1.jpg]

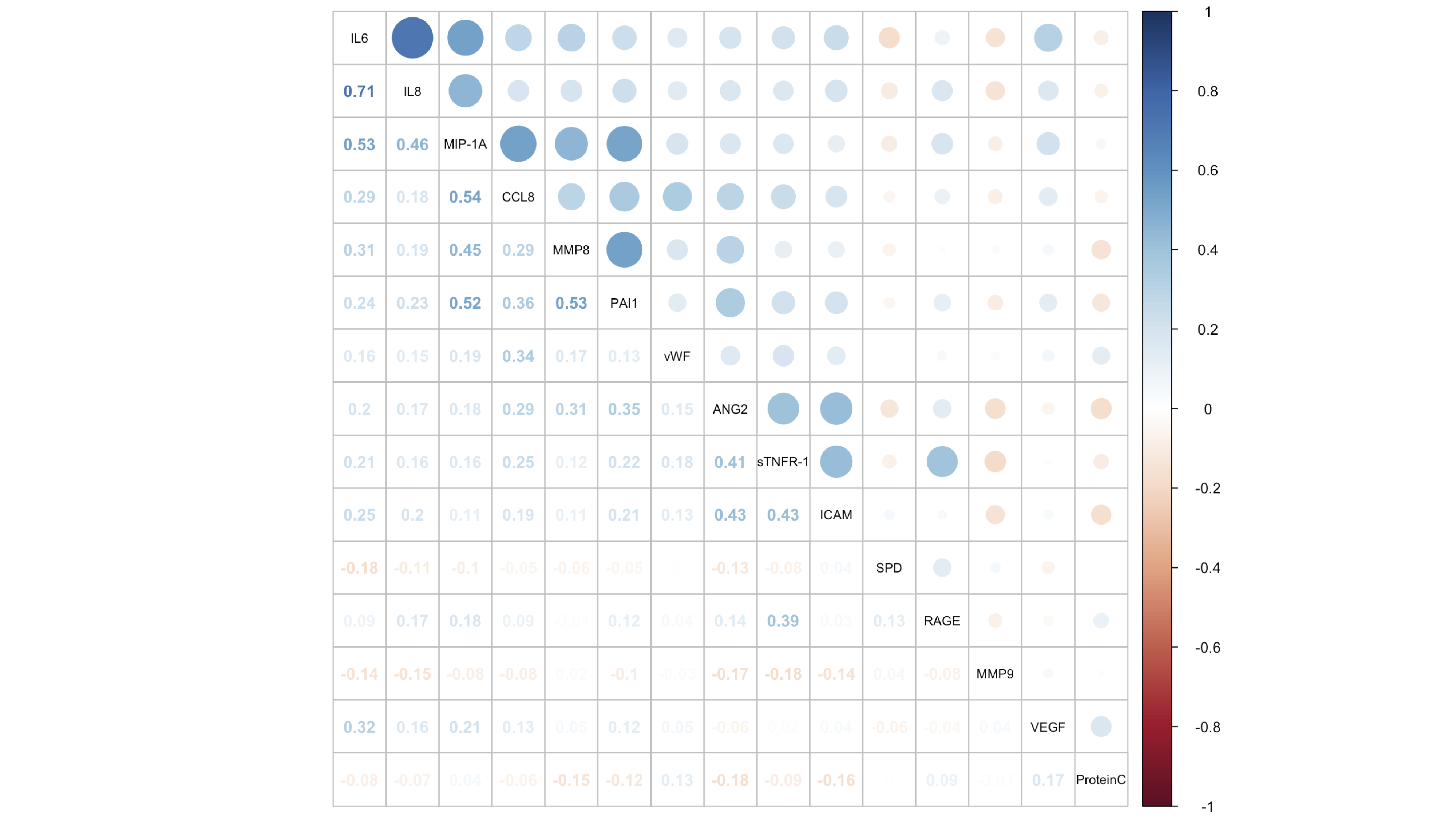

Supplement: FigureE6 [file NIHMS1753178-supplement-FigureE6.png]
