# Supplementary material for: Latent Class Analysis-Derived Subphenotypes are Generalizable to Observational Cohorts of Acute Respiratory Distress Syndrome: A Prospective Study
Source: Thorax. Author manuscript; Available in PMC 2023 Jan 1. (PMC8688287; doi:10.1136/thoraxjnl-2021-217158)
Supplement: Supplement Text [file NIHMS1753178-supplement-Supplement_Text.pdf]

Latent Class Analysis-Derived Subphenotypes are Generalizable to Observational Cohorts of Acute Respiratory Distress Syndrome: A Prospective Study (Supplementary Material)

Pratik Sinha[1], Kevin L Delucchi[2], Yue Chen[1,4], Hanjing Zhuo[3,4], Jason Abbott[3,4], Chunxue Wang[5], Nancy Wickersham[5], J. Brennan McNeil[5], Alejandra Jauregui[3,4], Serena Ke[3,4], Kathryn Vessel[3,4], Antonio Gomez[3,6], Carolyn M Hendrickson[3,6], Kirsten N Kangelaris[7], Aartik Sarma[3], Aleksandra Leligdowicz[3,8], Kathleen Liu[2,4,9], Michael A Matthay[3,4,10], Lorraine B Ware[5,11], and Carolyn S Calfee[3,4,10].

Affiliations:
1 Division of Clinical and Translational Research, Division of Critical Care, Department of Anesthesia, Washington University School of Medicine, Saint Louis, MO
2 Department of Psychiatry; University of California, San Francisco; San Francisco, CA
3 Department of Medicine, Division of Pulmonary, Critical Care, Allergy and Sleep Medicine; University of California, San Francisco; San Francisco, CA
4 Cardiovascular Research Institute; University of California, San Francisco; San Francisco, CA
5 Division of Allergy, Pulmonary, and Critical Care Medicine, Department of Medicine, Vanderbilt University Medical Center, Nashville, TN
6 Division of Allergy, Pulmonary, and Critical Care Medicine, Department of Medicine, University of California, Zuckerberg San Francisco General Hospital and Trauma Center
7 Division of Hospital Medicine, Department of Medicine, University of California, San Francisco; San Francisco, CA
8 Interdepartmental Division of Critical Care Medicine, University of Toronto, Toronto, Canada
9 Department of Medicine, Division of Nephrology, University of California, San Francisco; San Francisco, CA
10 Department of Anesthesia; University of California, San Francisco; San Francisco, CA
11 Department of Pathology, Microbiology and Immunology, Vanderbilt University Medical Center, Nashville, TN

Corresponding Author: Dr. Pratik Sinha
660 S. Euclid Ave, Campus Box 8054
St. Louis, MO 63110
Ph: 415-476-5756; email: p.sinha@wustl.edu

Methods

Participants Data collection

For both cohorts, the Validating of biomarker in Acute Lung Injury Diagnosis (VALID) study and the Early Assessment of Renal and Lung Injury (EARLI) study, consent was obtained, where possible, from patients or their surrogates. For patients that were unable to consent with no identified surrogates or if the patient died before they were approached for consent, a waiver of consent was provided.

In both cohorts, comprehensive data were collated into a database either by direct extraction from electronic medical records or manually by trained study coordinators. Adjudication of clinical subphenotypes was performed by critical care physicians, and ARDS was diagnosed after consensus of two board-certified physicians independently reviewing radiological findings and clinical data. The risk factors for ARDS were designated after careful review by the phenotyping physician.

Where available, the PaO_2/FiO_2 ratio was used to determine the presence of ARDS; otherwise, the SpO_2/FiO_2 ratio was used.[1] Severity scores were calculated using variables from the database. Different APACHE scores were used in the two studies; in VALID, the APACHE II score was available, and in EARLI, the APACHE III score was available. There were other key differences between the timing of recruitment of the two studies. For VALID, enrollment eligibility commenced on day 2 of ICU admission but data was collected for day 1 of ICU admission after enrollment. By contrast in EARLI, patients were eligible for enrollment once an ICU admission had been sought by the attending team in the Emergency Department. Finally, another major difference between the two cohorts was that VALID included patients with trauma as their primary aetiology for ARDS, whereas EARLI excluded such patients. The primary reason for this was that VALID was a single-center study performed at a Level I trauma center. In contrast, EARLI was performed at split sites with the primary data collection site (UCSF medical center) not a designated trauma center.

Biomarker Quantification

For both studies, after collection, blood samples were processed and plasma stored at -80°C. until batch quantification. Plasma samples frozen at baseline were thawed for protein biomarker quantification. Samples were frozen in small aliquots to minimize freeze-thaw cycles. For VALID, commercially available singleplex or multiplex ELISA assays were used for biomarker quantification: plasminogen activator inhibitor-1 (PAI-1) and protein C (Helena Laboratories; Beaumont, Tx), interleukin-8 (IL-8) and interleukin-6 (IL-6) (Meso Scale Diagnostics, LLC), intercellular adhesion molecule-1 (ICAM-1) and soluble tumor necrosis factor receptor-1 (sTNFR-1) (R&D systems; Minneapolis, MN). All analytes were measured in duplicate.

For EARLI, singleplex assays were used for the quantification of protein C (Helena Laboratories; Beaumont, TX) and plasminogen activator inhibitor-1 (PAI-1, R&D systems; Minneapolis, MN) and were measured as duplicates. All other biomarkers-- interleukin-8 (IL-8), interleukin-6 (IL-6), soluble tumor-necrosis factor receptor-1 (sTNFR-1), and intercellular adhesion molecule-1 (ICAM-1) -- were measured using a multiplex ELISA (R&D systems; Minneapolis, MN). The novel

research biomarkers were also quantified using multiplex ELISAs (R&D systems; Minneapolis, MN) and measured at the same time-point as the main set of biomarkers used for LCA. All multiplex assays were performed as single quantifications. For all biomarkers, a thorough quality control procedure was followed for each analysed plate.

Latent Class Analysis

Latent class analysis (LCA) is a form of mixture modelling that works on the assumption that concealed within a given multivariate distribution of a population there are unmeasured or 'latent' classes. Viewed simply, LCA is a model-based probabilistic method of clustering that identifies relatively homogeneous subgroups with a heterogeneous population. Further, unlike most other methods of clustering, LCA allows objective indices of model fitting such as the Bayesian Information Criteria (BIC), which in turn, allow statistical testing for the most optimal model for a given population.[2] The large sample size in both cohorts coupled with the known discriminatory properties of the variables in similar historical populations, we considered the study to be sufficiently powered to perform LCA in the primary analyses.[2] Among the secondary analyses, the EARLI cohort meeting the Berlin cohort may have been underpowered and represents a limitation of the study.

A combination of continuous and categorical variables was used for the LCA modelling. A list of the class-defining variables for each of the cohorts are listed in **Tables S2-6**. Observed variances between tables were due to data availability. Four models were built each consisting of one, two, three and four classes respectively. The best-fitting model for a given cohort was determined using the Bayesian Information Criteria (BIC), Vuong-Lo-Mendell-Rubin likelihood ratio (VLMR) test, entropy and the number of observations in the smallest class as previously. Once the optimal model was identified for a population, individual patients were assigned to a class based on their highest posterior probability. For the primary analysis in both studies (i.e. AECC cohorts, and AECC without trauma in VALID), a single variable for severity of hypoxemia was created such that SPO_2/FiO_2 was converted to equivalent PaO_2/FiO_2 using published algorithms where the latter was not available.[1] Due to a tendency towards extreme low values of the converted PaO_2/FiO_2 in patients with severe SPO_2/FiO_2 impairment, failure of oxygenation was converted into a categorical variable with three levels (mild < 300 mmHg and ≥ 200 mmHg; moderate < 200 mmHg and ≥ 100 mmHg; and severe < 100 mmHg). As a sensitivity analysis, PaO_2/FiO_2 was used as a continuous variable; the results were similar to those presented in terms of optimal classes and their composition, however, there were issues with model fitting with the more complex models failing to reproducibly converge to the maximum likelihood (3-class and 4-class models; data not presented). For the Berlin cohorts modelling, PaO_2/FiO_2 was used as a continuous variable. APACHE and SOFA scores were excluded from the model.

For the EARLI cohort, some protein biomarker levels were either greater than or less than the level of detection. For values greater than the level of detection, the value was replaced by the upper limit of detection (PAI-1: n = 2; IL6: n = 8; sTNFR-1: n = 6, IL-8: n = 8; Protein C: n = 1). For values lower than the level of detection (LLD), the values were replaced by the lower level of detection divided by 2 (LLD/2; sTNFR-1: n = 3, IL-8: n = 1). Sensitivity analyses were performed

with the level of detection set as missing and with the LLD set as its value (LLD); in these analyses, the results of the LCA remained unchanged (results not shown).

For the LCA modelling, all continuous variables underwent z-scale transformation. Additionally, non-normally distributed variables were log-transformed prior to z-scale transformation. Once the model with the optimal number of classes was determined for the population, average probabilities for class assignment were generated for each observation. A priori, in line with our previous work, patients were assigned to a subsubphenotype if the LCA-generated probability for that subphenotype was 0.5 or greater.

Parsimonious Classifier Models

In order to enable prospective evaluation of ARDS subphenotypes, logistic regression based parsimonious models comprising of 3 to 4 key predictor variables have been described that perform with high accuracy in randomized controlled trial (RCT) population.[3] Briefly, three National Heart, Lung, and Blood Institute ARDS network RCTs were used to develop the models and a fourth RCT was used to validate it. A 3-variable model comprising of IL-8, bicarbonate and Protein C, and a 4-variable model with the addition of vasopressor-use, were adjudicated as the best performing models. These two models were used for analyses in all described cohorts. LCA-derived subphenotype served as the reference standard to test model accuracy. Biomarker data were all log-transformed prior to application of the model. As with all logistic regression models, only cases where data were complete could be used for analysis. In VALID, all data were complete. In EARLI, data were available for 292 patients in the AECC cohort and 244 for the Berlin cohort.

References:

1. Rice TW, Wheeler AP, Bernard GR, Hayden DL, Schoenfeld DA, Ware LB, National Institutes of Health NHL, Blood Institute AN, (2007) Comparison of the SpO2/FIO2 ratio and the PaO2/FIO2 ratio in patients with acute lung injury or ARDS. Chest 132: 410-417
2. Sinha P, Calfee CS, Delucchi KL, (2021) Practitioner's Guide to Latent Class Analysis: Methodological Considerations and Common Pitfalls. Crit Care Med 49: e63-e79
3. Sinha P, Delucchi KL, McAuley DF, O'Kane CM, Matthay MA, Calfee CS, (2020) Development and validation of parsimonious algorithms to classify acute respiratory distress syndrome phenotypes: a secondary analysis of randomised controlled trials. Lancet Respir Med 8: 247-257

Tables

Table S1. Main exclusion criteria for the VALID and EARLI cohorts.

Exclusion Criteria for VALID	Exclusion Criteria for EARLI
ICU stay in outside hospital > 48 hours	Admission to trauma ICU
Uncomplicated overdose	Preference for comfort measure at ICU admission
Severe chronic lung disease	Isolated neurological or neurosurgical diagnosis
Likely transfer out of ICU on day 2	Expected to be in ICU < 24 hours
Post-surgical or non-intubated patients in cardiovascular ICU	Pregnant patients and prisoners
	Trauma as the primary admitting diagnosis

ICU = Intensive Care Unit

Table S2 Comparison of class defining variables between ARDS subphenotypes in the *VALID AECC* dataset.

Class defining variables for LCA model VALID AECC		Subphenotype 1 (n = 457)	Subphenotype 2 (n = 167)	P-value
**Age (years)**		52 (± 18)	55 (± 16)	0.16
**Sex: Female**		189 (41%)	72 (43%)	0.76[b]
**Race: White**		392 (86%)	152 (91%)	0.11[b]
**Race: Other**		65 (14%)	15 (9%)	
**Weight (kg)**		85 (± 27)	80 (± 25)	0.0338
**ARDS risk factor**	**Pneumonia**	110 (24%)	46 (28%)	< 0.0001[b]
	**Sepsis**	67 (15%)	81 (48%)	
	**Aspiration**	81 (18%)	27 (16%)	
	**Trauma**	161 (35%)	11 (7%)	
	**Other**	38 (8%)	2 (1%)	
**Temperature (°C)**		38 (± 0.9)	38 (± 1.6)	0.21
**Heart rate (beats.min^{-1})**		119 (± 21)	128 (± 21)	< 0.0001
**Systolic blood pressure (mmHg)**		89 (± 16)	81 (± 14)	< 0.0001
**Vasopressor use at baseline**		164 (36%)	104 (62%)	< 0.0001[b]
**Respiratory rate (breaths.min^{-1})**		28 (24 – 33)	34 (28 – 39)	< 0.0001[a]
**Hypoxia Category**	**Mild**	117 (26%)	38 (23%)	0.60[b]
	**Moderate**	194 (42%)	69 (41%)	
	**Severe**	146 (32%)	60 (36%)	
**$PaCO_2$ (mmHg)**		50 (± 13)	43 (± 12)	< 0.0001
**Tidal Volume (mL)**		444 (± 78)	431 (± 77)	0.08
**Positive end-expiratory pressure (cmH_2O)**		10 (8 – 12)	10 (8 – 13)	0.14[a]
**Hematocrit (%)**		30.4 (± 6)	27.5 (± 7)	< 0.0001
**White cell count ($10^3/\mu L$)**		16.6 (± 9)	14.3 (± 14)	0.06
**Platelets ($10^3/\mu L$)**		185 (± 107)	118 (± 113)	< 0.0001
**Sodium (mmol/L)**		140 (± 5)	138 (± 7)	0.0041
**Creatinine (mg/dL)**		1.5 (± 1.3)	2.4 (± 1.9)	< 0.0001
**Bicarbonate (mmol/L)**		22 (± 4)	19 (± 5)	< 0.0001
**Albumin (g/dL)**		2.7 (± 0.7)	2.5 (± 0.6)	0.0011
**Bilirubin (mg/dL)**		0.8 (0.6 – 1.2)	2.2 (1.2 – 5.9)	< 0.0001[a]
**Interleukin-6 (pg/mL)**		38 (17 – 86)	370 (116 – 1747)	< 0.0001[a]
**Interleukin-8 (pg/mL)**		11 (6 – 22)	143 (53 – 531)	< 0.0001[a]
**Soluble tumor-necrosis factor receptor-1 (pg/mL)**		1901 (1262- 3064)	4893 (3166 – 7157)	< 0.0001[a]
**Intercellular adhesion molecule 1 (ng/mL)**		341 (244 – 547)	753 (471 – 1083)	< 0.0001[a]
**Protein C (% control)**		74 (± 40)	44 (± 24)	< 0.0001
**Plasminogen activator inhibitor 1 (ng/mL)**		18 (7 – 33)	27 (15 – 47)	< 0.0001[a]

P values represent the 2-sample t-test unless annotated (a = Wilcoxon Rank Test, b = chi-square test). *AECC = American-European Consensus Conference.*

Table S3 Comparison of class defining variables between ARDS subphenotypes in the *EARLI AECC*

Class defining variables for LCA model EARLI AECC		Subphenotype 1 (n = 211)	Subphenotype 2 (n = 124)	P-value
**Age (years)**		66 (± 18)	66 (± 16)	0.82
**Sex: Female**		90 (43%)	57 (46%)	0.28[b]
**Race**	**White**	116 (55%)	49 (40%)	0.0146[b]
	**Black**	32 (15%)	17 (14%)	
	**Asian**	40 (19%)	40 (32%)	
	**Other**	23 (11%)	18 (15%)	
**Body mass index (kg/m^2)**		28 (± 11)	25 (± 8)	0.0029
**ARDS risk factor**	**Pneumonia**	106 (50%)	39 (31%)	< 0.0001[b]
	**Sepsis**	55 (26%)	63 (51%)	
	**Aspiration**	38 (18%)	13 (10%)	
	**Other**	12 (6%)	9 (7%)	
**Temperature (°C)**		37.7 (± 1.2)	37.8 (± 1.6)	0.31
**Heart rate (beats.min^{-1})**		120 (± 26)	136 (± 26)	<0.0001
**Systolic blood pressure (mmHg)**		92 (± 21)	76 (± 17)	< 0.0001
**Vasopressor use at baseline**		96 (45%)	116 (94%)	< 0.0001[b]
**Respiratory rate (breaths.min^{-1})**		33 (27 – 40)	35 (33 – 40)	0.0023[a]
**Hypoxia Category**	**Mild**	61 (29%)	23 (19%)	0.0232 [b]
	**Moderate**	82 (39%)	44 (35%)	
	**Severe**	68 (32%)	57 (46%)	
**$PaCO_2$ (mmHg)**		46 (± 17)	39 (± 15)	0.0003
**Minute ventilation (L.min^{-1})**		9.9 (± 3.3)	11.3 (± 3.2)	0.0127
**Positive end-expiratory pressure (cmH_2O)**		5 (5 – 10)	5 (5 – 8)	0.86[a]
**Hematocrit (%)**		32 (± 7)	28 (± 6)	< 0.0001
**White cell count ($10^3/\mu L$)**		14.1 (± 8.3)	17 (± 17.5)	0.13
**Platelets ($10^3/\mu L$)**		200 (± 100)	115 (± 91)	< 0.0001
**Sodium (mmol/L)**		136 (± 5)	134 (± 6)	0.0020
**Glucose (mg/dL)**		117 (± 42)	127 (± 94)	0.30
**Creatinine (mg/dL)**		1.3 (0.9 – 2.2)	1.9 (1.2 – 2.7)	< 0.0001[a]
**Bicarbonate (mmol/L)**		23 (± 5.12)	16 (± 4)	< 0.0001
**Albumin (g/dL)**		2.7 (± 0.7)	2.2 (± 0.7)	< 0.0001
**Bilirubin (mg/dL)**		0.9 (0.6 – 1.2)	1.3 (0.9 – 2.3)	< 0.0001[a]
**Interleukin-6 (pg/mL)**		83 (29 – 211)	4704 (551 – 11295)	< 0.0001[a]
**Interleukin-8 (pg/mL)**		16 (7 – 35)	323 (75 – 3372)	< 0.0001 [a]
**Soluble tumor-necrosis factor receptor-1 (pg/mL)**		3240 (1631 – 6093)	9394 (4736 – 15126)	< 0.0001 [a]
**Intercellular adhesion molecule 1 (ng/mL)**		529 (293 – 790)	1265 (506 – 1970)	< 0.0001[a]
**Protein C (% control)**		100 (± 66)	73 (± 67)	0.0008
**Plasminogen activator inhibitor 1 (ng/mL)**		5 (2.6 – 9.2)	35 (17 – 77)	< 0.0001 [a]

dataset.

P values represent the 2-sample t-test unless annotated (a = Wilcoxon Rank Test, b = chi-square test). *AECC = American-European Consensus Conference.*

Table S4 Comparison of class defining variables between ARDS subphenotypes in the *VALID Berlin* dataset.

Class defining variables for LCA model VALID Berlin		Subphenotype 1 (n = 366)	Subphenotype 2 (n = 134)	P-value
**Age (years)**		51 (± 18)	54 (± 16)	0.08
**Sex: Female**		147 (40%)	59 (44%)	0.50[b]
**Race: White**		308 (84%)	122 (91%)	0.07[b]
**Race: Other**		58 (16%)	12 (9%)	
**Weight (kg)**		86 (± 28)	79 (± 23)	0.0036
**ARDS risk factor**	**Pneumonia**	67 (18%)	36 (27%)	< 0.0001[b]
	**Sepsis**	48 (13%)	62 (46%)	
	**Aspiration**	65 (18%)	25 (19%)	
	**Trauma**	156 (43%)	11 (8%)	
	**Other**	30 (8%)	0 (0%)	
**Temperature (°C)**		38 (± 0.8)	38 (± 1.0)	0.17
**Heart rate (beats.min^{-1})**		120 (± 21)	130 (± 21)	< 0.0001
**Systolic blood pressure (mmHg)**		87 (± 15)	81 (± 14)	< 0.0001
**Vasopressor use at baseline**		146 (40%)	91 (68%)	< 0.0001[b]
**Respiratory rate (breaths.min^{-1})**		27 (22 – 31)	34 (28 – 39)	< 0.0001
**PaO_2/FiO_2**		143 (± 63)	133 (± 67)	0.16
**$PaCO_2$ (mmHg)**		52 (± 13)	44 (± 12)	< 0.0001
**Tidal Volume (mL)**		445 (± 78)	429 (± 74)	0.0428
**Positive end-expiratory pressure (cmH_2O)**		10 (8 – 12)	10 (8 – 12)	0.23 [a]
**Hematocrit (%)**		30.4 (± 7)	27.8 (± 7)	0.0001
**White cell count ($10^3/\mu L$)**		17 (± 9)	13.9 (± 13)	0.0123
**Platelets ($10^3/\mu L$)**		178 (± 99)	128 (± 116)	< 0.0001
**Sodium (mmol/L)**		140 (± 5)	138 (± 7)	0.0073
**Creatinine (mg/dL)**		1.5 (± 1.3)	2.4 (± 1.9)	< 0.0001
**Bicarbonate (mmol/L)**		22 (± 4)	19 (± 5)	< 0.0001
**Albumin (g/dL)**		2.8 (± 0.6)	2.4 (± 0.5)	< 0.0001
**Bilirubin (mg/dL)**		0.8 (0.6 – 1.2)	2.2 (1.2 – 4.9)	< 0.0001[a]
**Interleukin-6 (pg/mL)**		40 (18 – 94)	407 (122 – 1869)	< 0.0001[a]
**Interleukin-8 (pg/mL)**		11 (6 – 23)	148 (55 – 461)	< 0.0001 [a]
**Soluble tumor-necrosis factor receptor-1 (pg/mL)**		1833 (1194- 2889)	4991 (3174 – 7794)	< 0.0001 [a]
**Intercellular adhesion molecule 1 (ng/mL)**		324 (242 – 513)	696 (440 – 1106)	< 0.0001[a]
**Protein C (% control)**		73 (± 39)	45 (± 23)	< 0.0001
**Plasminogen activator inhibitor 1 (ng/mL)**		18 (9 – 35)	28 (16 – 50)	< 0.0001 [a]

P values represent the 2-sample t-test unless annotated (a = Wilcoxon Rank Test, b = chi-square test).

Table S5 Comparison of class defining variables between ARDS subphenotypes in the *EARLI Berlin* dataset.

Class defining variables for LCA model EARLI Berlin		Subphenotype 1 (n = 183)	Subphenotype 2 (n = 94)	P-value
**Age (years)**		65 (± 17)	66 (± 16)	0.78
**Sex: Female**		74 (40%)	40 (43%)	0.45[b]
**Race**	**White**	96 (53%)	42 (45%)	0.0077[b]
	**Black**	33 (18%)	7 (7%)	
	**Asian**	37 (20%)	28 (30%)	
	**Other**	17 (9%)	17 (18%)	
**Body mass index (kg/m^2)**		29 (± 11)	26 (± 8)	0.0181
**ARDS risk factor**	**Pneumonia**	85 (46%)	31 (33%)	0.0039[b]
	**Sepsis**	50 (27%)	46 (49%)	
	**Aspiration**	36 (20%)	11 (12%)	
	**Other**	12 (7%)	6 (6%)	
**Temperature (°C)**		37.6 (± 1.4)	37.7 (± 1.5)	0.62
**Heart rate (beats.min^{-1})**		121 (± 27)	138 (± 24)	<0.0001
**Systolic blood pressure (mmHg)**		90 (± 21)	73 (± 14)	< 0.0001
**Vasopressor use at baseline**		98 (53%)	92 (98%)	< 0.0001[b]
**Respiratory rate (breaths.min^{-1})**		33 (27 – 39)	35 (33 – 39)	0.0046[a]
**PaO_2/FiO_2 (mmHg)**		146 (± 65)	111 (± 64)	< 0.0001
**$PaCO_2$ (mmHg)**		46 (± 17)	42 (± 16)	0.07
**Minute ventilation (L.min^{-1})**		10.0 (± 3.2)	11.3 (± 3.4)	0.0326
**Positive end-expiratory pressure (cmH_2O)**		5 (5 – 8.5)	5 (5 – 8.5)	0.84[a]
**Hematocrit (%)**		32 (± 7)	28 (± 6)	< 0.0001
**White cell count ($10^3/\mu L$)**		14.6 (± 10)	15.5 (± 17.8)	0.66
**Platelets ($10^3/\mu L$)**		193 (± 96)	114 (± 95)	< 0.0001
**Sodium (mmol/L)**		136 (± 5)	133 (± 6)	< 0.0001
**Glucose (mg/dL)**		115 (± 43)	119 (± 59)	0.51
**Creatinine (mg/dL)**		1.3 (0.9 – 2.2)	1.9 (1.3 – 2.5)	0.0005[a]
**Bicarbonate (mmol/L)**		23 (± 6)	16 (± 4)	< 0.0001
**Albumin (g/dL)**		2.7 (± 0.6)	2.1 (± 0.6)	< 0.0001
**Bilirubin (mg/dL)**		0.9 (0.6 – 1.2)	1.3 (0.9 – 2.7)	< 0.0001[a]
**Interleukin-6 (pg/mL)**		93 (33 – 238)	6388 (1640 – 14610)	< 0.0001[a]
**Interleukin-8 (pg/mL)**		17 (8 – 38)	873 (192 – 3780)	< 0.0001[a]
**Soluble tumor-necrosis factor receptor-1 (pg/mL)**		3448 (1683 – 7572)	9468 (5032 – 14663)	< 0.0001[a]
**Intercellular adhesion molecule 1 (ng/mL)**		520 (301 – 790)	1315 (502 – 2073)	< 0.0001[a]
**Protein C (% control)**		100 (± 70)	74 (± 71)	0.0057
**Plasminogen activator inhibitor 1 (ng/mL)**		5 (2.6 – 13.4)	47 (19.9 – 81.7)	< 0.0001[a]

P values represent the 2-sample t-test unless annotated (a = Wilcoxon Rank Test, b = chi-square test).

Table S6 Comparison of class defining variables between ARDS subphenotypes in the *VALID AECC* dataset excluding trauma patients.

Class defining variables for LCA model VALID AECC		Subphenotype 1 (n = 315)	Subphenotype 2 (n = 137)	P-value
**Age (years)**		56 (± 17)	54 (± 15)	0.12
**Sex: Female**		153 (49%)	61 (45%)	0.49[b]
**Race**	**White**	270 (86%)	131 (96%)	0.0038[b]
	**Other**	45 (14%)	6 (4%)	
**Weight (kg)**		83 (± 28)	81 (± 26)	0.39
**ARDS risk factor**	**Pneumonia**	117 (37%)	39 (28%)	< 0.0001[b]
	**Sepsis**	74 (23%)	74 (54%)	
	**Aspiration**	86 (27%)	22 (16%)	
	**Other**	38 (12%)	2 (1%)	
**Temperature (°C)**		38 (± 1.0)	38 (± 1.0)	0.12
**Heart rate (beats.min^{-1})**		118 (± 21)	128 (± 20)	< 0.0001
**Systolic blood pressure (mmHg)**		89 (± 16)	83 (± 15)	0.0001
**Vasopressor use at baseline**		118 (37%)	81 (59%)	< 0.0001[b]
**Respiratory rate (breaths.min^{-1})**		30 (25 – 35)	34 (28 – 39)	< 0.0001[a]
**Hypoxia Category**	**Mild**	69 (22%)	31 (23%)	0.36[b]
	**Moderate**	119 (38%)	60 (44%)	
	**Severe**	127 (40%)	46 (34%)	
**$PaCO_2$ (mmHg)**		49 (± 15)	42 (± 12)	< 0.0001
**Tidal Volume (mL)**		424 (± 75)	432 (± 79)	0.42
**Positive end-expiratory pressure (cmH_2O)**		10 (8 – 12)	10 (8 – 13.5)	0.10[a]
**Hematocrit (%)**		30.6 (± 6)	27.6 (± 7)	< 0.0001
**White cell count ($10^3/\mu L$)**		15.9 (± 10)	14.3 (± 15)	0.27
**Platelets ($10^3/\mu L$)**		213 (± 118)	111 (± 105)	< 0.0001
**Sodium (mmol/L)**		139 (± 5)	138 (± 7)	0.11
**Creatinine (mg/dL)**		1.8 (± 1.7)	2.4 (± 1.8)	0.0006
**Bicarbonate (mmol/L)**		23 (± 5)	19 (± 5)	< 0.0001
**Albumin (g/dL)**		2.7 (± 0.7)	2.5 (± 0.6)	0.0020
**Bilirubin (mg/dL)**		0.9 (0.6 – 1.2)	2.7 (1.3 – 6.3)	< 0.0001[a]
**Interleukin-6 (pg/mL)**		31 (14 – 86)	370 (113 – 1908)	< 0.0001[a]
**Interleukin-8 (pg/mL)**		11 (6 – 23)	156 (61 – 663)	< 0.0001[a]
**Soluble tumor-necrosis factor receptor-1 (pg/mL)**		2261 (1585 – 3633)	5176 (3471 – 7947)	< 0.0001[a]
**Intercellular adhesion molecule 1 (ng/mL)**		441 (305 – 656)	825 (521 – 1147)	< 0.0001[a]
**Protein C (% control)**		71 (± 39)	41 (± 23)	< 0.0001
**Plasminogen activator inhibitor 1 (ng/mL)**		17 (6.4 – 32)	27 (15 – 50)	< 0.0001[a]

P values represent the 2-sample t-test unless annotated (a = Wilcoxon Rank Test, b = chi-square test). *AECC = American-European Consensus Conference.*

Table S7 Fit-statistics of the latent class analysis models for patients that met the AECC definition of ARDS in the VALID cohort with the trauma associated ARDS patients excluded.

Classes	BIC	VLMR p	N_1	N_2	N_3	N_4	Entropy
1	31396	–	452	–	–	–	–
2	30783	0.0129	315	137	–	–	0.85
3	30690	0.09	257	171	24	–	0.90
4	30682	0.67	250	125	55	22	0.91

BIC = Bayesian Information Criteria. VLMR = Vuong-Lo-Mendel-Rubin test. N represents the number of observations in each class. AECC = American-European Consensus Conference.

Table S8 Fit-statistics of the latent class analysis models for patients that met the Berlin definition of ARDS in the VALID cohort.

Classes	BIC	VLMR p	N_1	N_2	N_3	N_4	Entropy
1	35381	–	500	–	–	–	–
2	34673	0.0012	366	134	–	–	0.88
3	34513	0.53	204	182	114	–	0.90
4	34474	0.45	185	147	88	80	0.89

BIC = Bayesian Information Criteria. VLMR = Vuong-Lo-Mendel-Rubin test. N represents the number of observations in each class.

Table S9 Fit-statistics of the latent class analysis models for patients that met the Berlin definition of ARDS in the EARLI cohort.

Classes	BIC	VLMR p	N_1	N_2	N_3	N_4	Entropy
1	20393	–	277	–	–	–	–
2	19863	0.0497	183	94	–	–	0.88
3	19762	0.11	147	68	62	–	0.88
4	19787	0.76	118	70	48	41	0.88

BIC = Bayesian Information Criteria. VLMR = Vuong-Lo-Mendel-Rubin test. N represents the number of observations in each class.

Table S10. ***Ac*****ute Physiology and Chronic Health Evaluation (APACHE) II scores in the primary and secondary cohorts stratified by ARDS subphenotypes.**

	Hypoinflammatory	Hyperinflammatory	P-Value
**VALID AECC**	**26 ± 7**	**33 ± 7**	**< 0.0001**
**VALID AECC (NO TRAUMA)**	**27 ± 8**	**33 ± 7**	**< 0.0001**
**EARLI AECC**	**27 ± 8**	**36 ± 9**	**< 0.0001**
**VALID Berlin**	**27 ± 7**	**35 ± 7**	**< 0.0001**
**EARLI Berlin**	**28 ± 8**	**38 ± 9**	**< 0.0001**

P- value represent the Student t-test. AECC = American-European Consensus Conference.

Table S11 Clinical outcomes in the Berlin cohorts stratified by ARDS subphenotypes.

	Ventilator-Free Days			In-hospital mortality*		
	Hypo-inflammatory	Hyper-inflammatory	P-Value	Hypo-inflammatory	Hyper-inflammatory	P-Value
**VALID Berlin**	19 (11 – 24)	5 (0 – 20)	< 0.0001	61/366 (17%)	65/134 (49%)	< 0.0001
**EARLI Berlin**	23 (0 – 26)	0 (0 – 19)	< 0.0001	59/183 (32%)	66/94 (70%)	< 0.0001

*P- value represent Wilcoxon rank sum test for ventilator free days and chi-squared test for in hospital mortality. *Followed up to day 60 in EARLI and to discharge in VALID*

Table S12 Differences in indices of cardiac function and injury between the two subphenotypes.

	Data Availability	Median (IQR)	P-Value
**Left Ventricular Ejection Fraction (%; VALID only)[a]**			
Total Population	243/624 (39%)	55 (55-60)	--
Hypoinflammatory	160/457 (35%)	55 (55 – 56)	0.71
Hyperinflammatory	83/167 (50%)	55 (50 – 60)	
**Troponin I (ng/mL; EARLI only)[b]**			
Total Population	260/335 (78%)	0.06 (0.02 – 0.28)	--
Hypoinflammatory	159/211 (75%)	0.05 (0.02 – 0.15)	0.18
Hyperinflammatory	101/124 (81%)	0.09 (0.02 – 0.34)	
**Brain Natriuretic Peptide (pg/mL; EARLI only)[b]**			
Total Population	125/335 (37%)	259 (149 – 933)	--
Hypoinflammatory	87/211 (41%)	245 (162 – 923)	0.87
Hyperinflammatory	38/124 (31%)	282 (128 – 872)	

a = Recorded anytime during hospital admission. b = highest values during the first 5 days of study enrollment. P- value represent Wilcoxon rank sum test.

Table S13 Comparison of novel biomarkers between the two ARDS subphenotypes in EARLI AECC cohort.

	Hypoinflammatory (n = 211)		Hyperinflammatory (n = 124)		
**Biomarker**	**Missing**	**Median (IQR)**	**Missing**	**Median (IQR)**	**P-value**
**Ang-2 (pg/mL)**	24 (11%)	5242 (3136 – 9466)	11 (9%)	12204 (6507 – 23115)	< 0.0001
**CCL-8 (pg/mL)**	66 (31%)	40 (22 – 77)	37 (30%)	147 (54 – 315)	< 0.0001
**MIP-1α (pg/mL)**	65 (31%)	101 (101 – 192)	37 (30%)	223 (129 – 333)	< 0.0001
**MMP-8 (ng/mL)**	65 (31%)	55 (16 – 125)	37 (30%)	221 (103 – 552)	< 0.0001
**MMP-9 (ng/mL)**	66 (31%)	559 (216 – 1193)	37 (30%)	145 (48 – 385)	< 0.0001
**RAGE (pg/mL)**	24 (11%)	3342 (1892 – 6291)	11 (9%)	5097 (3207 – 8555)	< 0.0001
**SP-D (ng/mL)**	66 (31%)	94 (46 – 200)	37 (30%)	49 (28 – 151)	0.0011
**VEGF (pg/mL)**	65 (31%)	32 (16 – 57)	37 (30%)	32 (23 – 60)	0.07
**vWF (pg/mL)**	61 (29%)	90 (46 – 224)	35 (28%)	182 (68 – 325)	0.0055

ANG2: Angiopoietin-2; CCL8: Chemokine Ligand-8; MIP-1α: Macrophage Inflammatory Protein-1-alpha; MMP: Matrix Metalloproteinase; RAGE: Receptor for Advanced Glycation End-products; SPD: Surfactant Protein D; VEGF: Vascular Endothelial Growth Factor, vWF: von Willebrand Factor. P-value represents the Wilcoxon rank sum test. *AECC: American-European Consensus Conference.*

Figures

Figure S1. Flow diagram showing the screening and selection of patients extracted from the two observational cohorts for this study. A: VALID cohort. B: EARLI cohort.

Figure S2 Proportion of vasopressor-use in the two subphenotypes in the primary cohorts (* p < 00001).**

Figure S3. Comparison of hypoxia categories and Invasive ventilation between subphenotypes. Chi-square test was used to compare differences between subphenotypes (significant value denoted). **A: VALID AECC** (hypoxia categories p = 0.60; Invasive ventilation p = 0.94)**. B: VALID AECC with trauma patients excluded** (hypoxia categories p = 0.36; Invasive ventilation p = 0.29)**. C: EARLI AECC** (*p = 0.023, **p = 0.012)**.** AECC= American-European Consensus Conference.

Figure S4. Kaplan-Meier survival plots censored at day 60 stratified by ARDS subphenotypes A. VALID; B. EARLI

Figure S5. Receiver operating characteristic curves for the parsimonious classifier models in the EARLI AECC (n = 292) and Berlin Cohorts (n = 244). A: 3-variable model (interleukin-8, serum bicarbonate and protein c)**. B: 4-variable model** (interleukin-8, serum bicarbonate, protein c and vasopressor-use to the 3-variable model)**.** AUC: Area under the curve.

Figure S6. Correlation matrix of all plasma biomarkers quantified for the study in the EARLI cohort. IL: Interleukin, MIP-1A: Macrophage Inflammatory Protein-1-alpha, CCL8: Chemokine Ligand-8, MMP: Matrix Metalloproteinase, PAI1: Plasminogen Activator Inhibitor-1, vWF: von Willebrand Factor, ANG2: Angiopoietin-2, sTNFR1: soluble Tumor Necrosis Factor-1, ICAM: Intercellular Adhesion Molecule-1, SPD: Surfactant Protein D, RAGE: Receptor for Advanced Glycation End-products, VEGF: Vascular Endothelial Growth Factor.
